# ChatGPT for Automated Qualitative Research: Content Analysis

**DOI:** 10.2196/59050

**Published:** 2024-07-25

**Authors:** Rimke Bijker, Stephanie S Merkouris, Nicki A Dowling, Simone N Rodda

**Affiliations:** 1 Department of Psychology and Neuroscience Auckland University of Technology Auckland New Zealand; 2 School of Psychology Deakin University Burwood Australia

**Keywords:** ChatGPT, natural language processing, qualitative content analysis, Theoretical Domains Framework

## Abstract

**Background:**

Data analysis approaches such as qualitative content analysis are notoriously time and labor intensive because of the time to detect, assess, and code a large amount of data. Tools such as ChatGPT may have tremendous potential in automating at least some of the analysis.

**Objective:**

The aim of this study was to explore the utility of ChatGPT in conducting qualitative content analysis through the analysis of forum posts from people sharing their experiences on reducing their sugar consumption.

**Methods:**

Inductive and deductive content analysis were performed on 537 forum posts to detect mechanisms of behavior change. Thorough prompt engineering provided appropriate instructions for ChatGPT to execute data analysis tasks. Data identification involved extracting change mechanisms from a subset of forum posts. The precision of the extracted data was assessed through comparison with human coding. On the basis of the identified change mechanisms, coding schemes were developed with ChatGPT using data-driven (inductive) and theory-driven (deductive) content analysis approaches. The deductive approach was informed by the Theoretical Domains Framework using both an unconstrained coding scheme and a structured coding matrix. In total, 10 coding schemes were created from a subset of data and then applied to the full data set in 10 new conversations, resulting in 100 conversations each for inductive and unconstrained deductive analysis. A total of 10 further conversations coded the full data set into the structured coding matrix. Intercoder agreement was evaluated across and within coding schemes. ChatGPT output was also evaluated by the researchers to assess whether it reflected prompt instructions.

**Results:**

The precision of detecting change mechanisms in the data subset ranged from 66% to 88%. Overall κ scores for intercoder agreement ranged from 0.72 to 0.82 across inductive coding schemes and from 0.58 to 0.73 across unconstrained coding schemes and structured coding matrix. Coding into the best-performing coding scheme resulted in category-specific κ scores ranging from 0.67 to 0.95 for the inductive approach and from 0.13 to 0.87 for the deductive approaches. ChatGPT largely followed prompt instructions in producing a description of each coding scheme, although the wording for the inductively developed coding schemes was lengthier than specified.

**Conclusions:**

ChatGPT appears fairly reliable in assisting with qualitative analysis. ChatGPT performed better in developing an inductive coding scheme that emerged from the data than adapting an existing framework into an unconstrained coding scheme or coding directly into a structured matrix. The potential for ChatGPT to act as a second coder also appears promising, with almost perfect agreement in at least 1 coding scheme. The findings suggest that ChatGPT could prove useful as a tool to assist in each phase of qualitative content analysis, but multiple iterations are required to determine the reliability of each stage of analysis.

## Introduction

### Background

Emerging from a variety of fields such as psychology, nursing, media communication, and market research, content analysis has become one of the main methods for analyzing large qualitative data sets [1,2]. Content analysis uses systematic and replicable methods to synthesize and describe patterns in textual, visual, or audio data and facilitate understanding of the content and meaning of the data [1-4]. It can be used to analyze qualitative data obtained from interviews, focus groups, and patient records as well as naturally occurring data such as user-generated website content, discussion forums, social media, and customer databases [4,5]. Furthermore, content analysis can use quantitative methods, which focus on quantification of the data to enable calculation of prevalence data or use in statistical analyses, or qualitative methods, which focus on distilling concepts or constructs from the data to facilitate understanding [6]. Qualitative content analysis also allows for the integration of quantitative components, for instance, by summarizing the findings in frequency distributions, which can be particularly useful when working with large data sets [7].

Content analysis can be performed using an inductive, data-driven approach or a deductive, theory-driven approach. An inductive approach is suitable when the topic of interest is still emerging or lacking a firm scientific knowledge database [4,8,9]. In contrast, a deductive approach is more appropriate when there is an existing body of knowledge and the aim is to confirm hypotheses and code data into an existing framework or theory [3,10]. While the practice and philosophy of qualitative content analytic approaches may vary, they have similar tasks to be executed as part of the data analysis process, including identification of relevant data, organization of data, and data classification [6]. The systematic nature of this process provides opportunities for computer assistance. In fact, computer-assisted execution of qualitative analysis tasks has been around for some time with the aim of improving the speed and accuracy of the analysis process, especially when large data sets are involved [11].

Whether inductive or deductive, each approach to content analysis requires a preparation phase, which involves reading the text for familiarization and identifying the meaning units (ie, the words or sentences in the text that are relevant to the research questions and contained within the unit of analysis) by assigning them initial labels that reflect the key content of the meaning units [12]. It is recommended that this is repeated multiple times, whereby there is a cycle of reading and rereading the data and adjusting the labels so that they represent the data that are being coded [1]. The data are then subject to condensation, where the meaning units are summarized into short descriptions that maintain the key content of the data and are assigned codes that are grouped according to content similarity [13]. Traditional qualitative analysis software offers automated coding, but multiple studies have reported inaccuracies, suggesting that manual coding should remain the dominant approach [13,14].

Following the preparation phase, machine learning may assist in the development of a coding scheme (also called a coding frame or data dictionary) that can be used to systematically organize the data by allocating each code to that scheme. When the analysis involves large data sets, coding schemes are typically constructed on a subset of data, after which the coding scheme can be applied to the full data set [15]. For the inductive content analysis approach, the development of a coding scheme involves organizing codes and initial groupings into categories according to the research question [3,4]. Categories are continuously adjusted and are often split into subgroups to ensure that they are discrete and mutually exclusive while still adequately reflecting the data [12]. Meaning units might have to be adapted as well to ensure that each code belongs to only 1 category [2]. Multiple researchers can also develop lists of categories that are combined into 1 coding scheme via negotiation [16]. For each category, labels are developed to define the category, and commonly, a separate coding manual is composed to specify coding rules [2].

For the deductive content analysis approach, codes are similarly organized into categories; however, the categories are predefined in a coding matrix that is informed by a theory or framework [3,4]. The matrix can then be made into a coding scheme that reflects the data being coded by labeling and relabeling the categories in line with the current data set [3]. This unconstrained deductive approach results in a coding scheme in which different categories may emerge that still reflect the data and are consistent with the theory or framework. Alternatively, a structured deductive approach can test categories, concepts, and hypotheses by coding data directly into predefined categories of the coding matrix [3]. When using a structured coding matrix, the categories are not redefined to reflect the data being coded, thereby giving the researcher the opportunity to focus on the degree of alignment between the matrix and the current data set [3].

Whether following an inductive or unconstrained deductive approach, the development of a coding scheme is an iterative process that involves continuously updating the coding scheme until a version is achieved that can be used to reliably code the entire data set [17]. The development and refinement of coding schemes is generally conducted by the research team and may involve either others with expertise in the framework or theory or a literature review [3,4]. There is recent evidence suggesting that machine learning and natural language processing techniques are promising for relatively straightforward tasks such as data extraction and classification of unstructured qualitative data [18,19]. However, using such techniques typically requires programming skills that are unavailable to most researchers.

After the development phase, the quality of the coding schemes can be evaluated by assessing the reliability of application. This phase involves ≥2 researchers classifying the data into the defined categories and comparing the resulting annotated data sets [2]. Manual coding may be aided by technology in terms of sorting and renaming categories (eg, using Excel [Microsoft Corp] or qualitative analysis software), but this is also prone to fatigue and error, especially in cases in which coding schemes have multiple categories and subcategories or interpretation of meaning is part of the selected approach [3,10,13,20,21]. For example, reliability is dependent on the number of correctly coded items that may be at the higher order or subcategory level. While percentage of agreement is often reported [21], a more robust approach is through κ interrater agreement (henceforth referred to as intercoder agreement, a more appropriate term when classification relates to nominal categories) [22]. This intercoder agreement determines the degree to which the coders agree beyond what would be expected from chance alone [23]. The calculation of the κ score relies on each category in the coding scheme being mutually exclusive and the unit of analysis being independent in that it contains only 1 statement or code [23]. In cases in which the κ score indicates low intercoder agreement, the coding scheme might require further refinement until the application of the coding scheme achieves high intercoder agreement [2]. The final coding scheme can be used to classify the content of the full data set and produce frequency data for further analysis. In cases in which previous evaluation of the coding scheme was based on a data subset, reliability might be assessed again once the full data set has been annotated by multiple researchers as coding of large data sets may be prone to errors due to researcher fatigue and subtle changes in interpretation over time [2,6,13]. While reliability is commonly evaluated by intercoder agreement, an alternative option is intracoder agreement, in which the same person codes the same data on 2 separate occasions [24].

In addition to evaluating reliability, the validity of the findings from content analysis can be enhanced via triangulation, which can be achieved by combining methodologies, involving various investigators, or incorporating multiple theories [4,8,13,20,25]. In content analysis, increased validity might be achieved through transparency in reporting and potential for replication, confirmation that the categories relate to the research questions, member checks, evaluation of the appropriateness of the coding scheme by content experts, and the use of different data sources [3,4,13]. Other considerations relate to credibility (ie, how well data analysis procedures ensure the inclusion of all relevant data, which may be improved through consensus among the research team or external experts) and dependability (ie, how the analytic process may alter data over time—such as recoding data—thereby implying a need for careful logging of changes and consistent decision-making) [13].

### ChatGPT

Advances in artificial intelligence (AI) and machine learning technologies have greatly impacted working life by automating manual processes and assisting in laborious tasks. One of the more prominent developments by OpenAI is ChatGPT, a large language model (LLM) that has been trained using a wide range of data, which enables it to synthesize human inputs and generate humanlike responses [26]. Inputs are provided in the form of “prompts,” which contain questions or instructions that are processed to provide responses in line with the training data. According to an investigation by Altmetric, a platform that tracks web-based attention to research, the interest in ChatGPT appears to be particularly high among scientists [27]. Since its launch in November 2022 [28], scientists have enlisted the assistance of ChatGPT for a range of tasks, including conceptualizing ideas, summarizing scientific literature, generating analytic code, and writing up manuscripts [22,29]. In this field, ChatGPT’s potential has primarily been discussed in relation to scientific writing and editing. For example, ChatGPT has been used to conduct rapid literature reviews, translate and edit texts, structure manuscripts to increase readability and fulfill journal guidelines, write logically sound abstracts, and provide suggestions on how to address reviewer comments [26,30-32].

Research suggests that ChatGPT may be a promising tool for qualitative analysis methods [33-36]. As a natural language processing tool that applies computation techniques and semantic similarities to analyze and synthesize natural language and speech [37], ChatGPT may enhance the streamlining of tasks and increase the efficiency of qualitative research projects. Nevertheless, interviews with those involved in qualitative analysis suggest that the time needed for prompt engineering, understanding complex responses, and organizing unstructured output, combined with concerns about the accuracy and validity of the output, may not outweigh the benefits of incorporating ChatGPT as a tool in qualitative research [38].

### Study Aims

The aim of this study was to explore the utility of ChatGPT in conducting qualitative content analysis through the analysis of forum posts from people sharing their experiences on reducing their sugar consumption. Specifically, given the various analytic approaches that can be used, the aim was to explore the utility of ChatGPT in both inductive (ie, data-driven) and deductive (ie, using an existing framework) approaches. For the deductive approach, we used the Theoretical Domains Framework (TDF) as this is a commonly used framework to guide evaluation of the implementation of evidence-based practice in health care settings [39]. A secondary aim was to provide insights into the mechanisms of sugar reduction most frequently discussed in forum data by providing frequency data generated using the inductive and deductive approaches.

## Methods

### Overview

**Figure 1:**
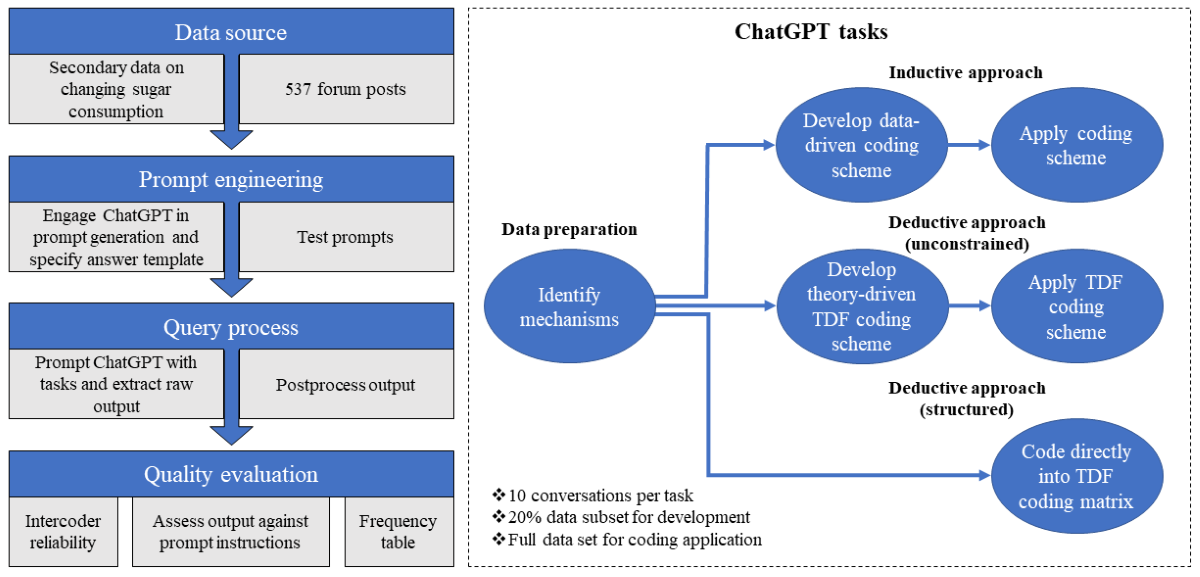
Study flow. TDF: Theoretical Domains Framework.

An overview of the study methods is presented in Figure 1. The qualitative content analysis process was broken down into several tasks—referred to as queries—that we identified as having the potential for automation using ChatGPT. Prompt engineering was used to create appropriate prompts with instructions in line with the respective tasks (ie, the queries). In an approximation of a manual coding process, ChatGPT was first used to generate a data set of condensed meaning units that were extracted from forum posts on sugar reduction. On the basis of a subset of the generated data set, ChatGPT was then used to create 10 versions of an inductive, data-driven coding scheme and 10 versions of an unconstrained deductive, theory-driven coding scheme. The unconstrained deductive coding scheme was based on the TDF. Thus, different versions of the coding schemes were developed in parallel, in contrast to a manual process in which coding schemes are developed iteratively and reflect more refined updates from previous versions. ChatGPT was then used to annotate the full data set by applying the coding schemes. ChatGPT was also used to code the full data set into a structured coding matrix based on the TDF, in which data were coded directly into the predefined categories guided only by the training data of ChatGPT’s underlying LLM. All coding scheme versions and the coding matrix were applied to the full data set 10 times in 10 new conversations, whereby each conversation was considered an independent coder. By doing so, the resulting annotated data sets could be compared to assess the interrater reliability of the coding schemes.

All ChatGPT queries were conducted from June 12 to 18, 2023, when ChatGPT operated under the GPT-3.5 Turbo model [40]. We opted to use the ChatGPT web application with unpaid subscriptions to ensure that our approach can be applied by anyone interested in using qualitative content analysis regardless of the programming skills and financial resources they have available to conduct their research.

### Data Source

The data source of this study was a study on mechanisms of sugar reduction that has been previously described [41]. Briefly, Google searches were conducted to identify web-based content in which internet users mentioned mechanisms related to changing sugar consumption. In this search, potentially relevant posts were sourced from consumer platforms (eg, forums and message boards); popular media platforms (eg, blogs, web-based magazines, and news articles); and professional platforms (governmental and treatment provider websites). User-generated posts (including context, where applicable) were manually transferred to a Microsoft Excel spreadsheet and assigned labels and codes reflecting the change mechanisms identified in the forum posts. Mechanisms were then analyzed to create an overview of the range of change mechanisms applied to reduce sugar consumption. For our study, we retained all consumer posts, which resulted in a data set with 539 unique forum posts.

### Ethical Considerations

All data were from open access sources and freely available without sign-in requirement or agreement with a specified set of terms and conditions. The retained posts did not contain any user-identifiable data. As such, this study was exempted from ethics approval in accordance with Auckland University of Technology Ethics Committee guidelines [42].

### Prompt Engineering

Research has highlighted the importance of using high-quality prompts for LLMs given that different prompts that appear to reflect similar instructions may lead to highly variable responses [43,44]. Therefore, 2 researchers (RB and SSM) engaged in a systematic process of prompt engineering to generate and compare prompts. To enhance the quality of our prompts, we used a combination of various techniques that have been suggested to improve responses in conversations with LLMs such as ChatGPT [45-47]. For example, we started conversations with prompts that included information on the context of our study and then applied techniques to engage ChatGPT in an iterative prompt engineering process in which instructions referred to writing relevant prompts, providing alternative prompts, refining prompts based on additional information, listing the pros and cons of prompts, and recommending prompts given the relevant pros and cons. Through this process, we refined and selected at least 3 prompts per analysis task that were all run several times to assess which prompts contained the most optimal instructions for use in the qualitative content analysis query process. Assessment at this stage was based on the extent to which responses were in line with the prompt instructions, such as correct response format, no apparent issues in response content, and comprehensiveness of the response.

The final set of prompts for the query process had a similar construction in that each prompt started with (1) detailed instructions on the task to be executed, including notes on what to watch out for, if applicable, followed by (2) specifications on the required response format or answer template and ending with (3) information on which the response had to be based (ie, list of forum posts or change mechanisms). In some prompts, the instructions followed a step-by-step structure [48] as prompt engineering showed that this resulted in a more comprehensive response. Textbox 1 provides a prompt example (with a step-by-step structure), and Multimedia Appendix 1 provides a complete overview of the final prompts. At the time of this research, ChatGPT had a limit on the number of tokens (ie, units that are meaningful to an LLM, such as words, word fragments, or punctuation signs) processed in each combined prompt and response. Consequently, for each query, we reprompted ChatGPT with the same instructions but different subsets of the data on which responses had to be based until all data were processed.

**Example of a prompt used in this study.**

**Example**

- Below are forum posts reflecting real-life experiences related to changing sugar consumption. For each of these posts, please extract all potential mechanisms, strategies, or techniques for reducing sugar consumption. Each mechanism should be described in a brief and concise manner, using up to 10 words. If multiple mechanisms can be extracted from a single post, each should be mentioned separately. Please note that mechanisms may not be explicitly presented as such, so be sure to interpret and extract them from the forum posts accordingly.
- First format the response as:
- “Post 1:
  mechanism 1: {max 10 word description}
  mechanism 2: {max 10 word description}
  etc (if applicable)
- Post 2:
  mechanism 1: {max 10 word description}
  mechanism 2: {max 10 word description}
  etc (if applicable)”
- Then please reformat the response as an excel spreadsheet with in the first column the post number, the second the mechanism number, and the third the mechanism description. Please format the table as csv and put the table in code block.
- Forum posts: “”*[insert forum posts]*“”

After prompt engineering, 4 new OpenAI accounts were created and used on 4 different computers to run the large number of queries. As such, the accounts were used to conduct tasks on a different computer simultaneously or repeat tasks on the same computer within an interval of 48 hours. New conversations were started for each task and ended when all data pertaining to the task in the query were processed (ie, 1 run of the task). Conversations were logically labeled to enable ease of lookup at a later stage (eg, Task1_account1_run3 and Task5_account4_run1).

### Query Process

#### Data Preparation

Data preparation involved creating a data set of condensed meaning units based on the change mechanisms identified in the forum posts. Posts from the original published sugar study were randomized using a random number generator function in Microsoft Excel. The forum posts were included alongside step-by-step instructions to identify potential change mechanisms from the provided posts (Textbox 1). The first step included directions to extract the mechanisms for reducing sugar consumption from the posts and summarize the change mechanisms in a brief description (up to 10 words). The resulting brief descriptions functioned as the condensed meaning units to be used for coding scheme development. In the next step, ChatGPT was instructed to reformat the response into a table with post number, mechanism number (within the post), and the brief description of each identified change mechanism. All output tables in the conversation were transferred to a Microsoft Excel spreadsheet, which received a label consistent with the conversation name. The data preparation query was repeated to emulate a manual data preparation phase involving multiple coders and enabling comparison of brief descriptions across coded data sets. The query was repeated 10 times in 10 different conversations, thereby resulting in 10 data sets reflecting 10 different coders.

Comparison of the 10 data sets generated by ChatGPT with human coding was based on the change mechanisms identified in 108 forum posts (ie, a 20% data subset). In total, 3 researchers (RB, SSM, and SNR) compared the condensed meaning units with the forum posts and indicated which condensed meaning units correctly reflected a change mechanism described in the forum posts and which condensed meaning units incorrectly identified a change mechanism from the posts. Data sets were double coded, and disagreements were discussed until consensus was achieved. The data set with the highest percentage of correctly identified change mechanisms across all identified change mechanisms (ie, the best precision) was selected for further use in the analysis. Specifically, the 20% data subset was used for the development of the inductive and unconstrained deductive coding schemes, after which the developed coding schemes were applied to the full data set of change mechanisms. For the structured deductive approach, the full data set was coded directly into a TDF coding matrix. Change mechanisms in the selected data set were given a unique identifier from 1 to *n* to enable data linkage in subsequent tasks.

#### Inductive Approach: Development and Application of Data-Driven Coding Schemes

The inductive approach started with a task that reflected the development of a coding scheme where categories are derived from the data [3]. The prompt developed for this task instructed ChatGPT to organize meaning units from the subset of data (20%) into categories based on underlying patterns in the data. The prompt included explicit instructions to ensure that there was no overlap between the categories. Furthermore, the instruction and answer template indicated that ChatGPT had to provide a 20-word label to define each category. The task was repeated in 10 new conversations to create 10 versions of an inductively developed coding scheme.

The next task in the inductive approach reflected the classification of the full data set into the inductively developed coding scheme. The output from the previous task was inserted into a prompt in which instructions specified that change mechanisms were to be classified under the best-matching category in accordance with the 20-word category definitions. ChatGPT was also instructed to format the output as a table that listed the mechanism unique identifier, the brief description of the change mechanism, and the best-matching category from the coding scheme. As there were 10 inductively developed coding schemes, the standard prompt was also adapted 10 times to reflect the various versions of the coding scheme. To reflect a process with 10 independent coders, each coding scheme was applied 10 times by starting 10 new conversations per coding scheme.

#### Unconstrained Deductive Approach: Development and Application of Theory-Driven Coding Schemes

The deductive approach started with the development of an unconstrained coding scheme that was informed by the TDF. The prompt instructed ChatGPT to identify domains (akin to categories in the inductive approach) from the TDF and redefine these domains to reflect the current data set subset. For the first step, the instructions specified grouping all mechanisms under the best-aligning domain, with explicit statements that a group could only reflect 1 domain and that each change mechanism could only be listed once. The instructions also specified listing each change mechanism under the domain in which it was grouped to ensure that each mechanism was listed once. Prompt engineering illustrated that although ChatGPT training data included sources on the TDF, prompt responses tended to incorporate fabricated or adapted domain names. Therefore, the prompt included a list of all 14 domains to remove any ambiguity in the instructions. For the second step, ChatGPT was instructed to provide a 20-word definition for each domain based on the group of change mechanisms it listed under the domain in the first step. The instructions further detailed that domains not identified in step 1 should be labeled as “N/A.” The task was repeated in 10 new conversations to create 10 versions of a deductively developed coding scheme.

The next task in the deductive approach reflected classification of the full data set into the domains of the deductively developed unconstrained coding scheme. The prompt instructed ChatGPT that change mechanisms were to be classified under the best-matching category in accordance with the 20-word category definitions. There was no reference to the TDF in the instructions to ensure that ChatGPT based its response on each provided coding scheme and to minimize the risk of unintentionally infusing the instructions with additional information based on ChatGPT’s preexisting knowledge of the TDF. The prompt was adapted 10 times (ie, once for each of the unconstrained coding schemes), and 10 new conversations were started per coding scheme (to mimic a process with 10 coders).

#### Structured Deductive Approach: Coding Directly Into TDF Coding Matrix

The full data set was classified directly into the domains of the TDF using a coding matrix that listed all 14 TDF domains [39] but was empty of definitions for the domains. Thus, this approach did not consider the underlying data and solely relied on ChatGPT’s preexisting knowledge of the TDF and how it would describe the mechanisms of sugar reduction. The prompt included instructions to classify each change mechanism under only the TDF domain that best matched each mechanism and specified the response format to be in the form of a table with columns for the mechanism unique identifier, short description, and the best-matching TDF domain. Direct coding into the TDF was conducted over 10 new conversations, reflective of a process with 10 coders.

#### Postprocessing

The output of conversations applying the coding schemes to the full data set was manually transferred to Microsoft Excel spreadsheets and labeled in accordance with the conversation names. As described previously [49], ChatGPT-generated data commonly require various postprocessing steps to clean the data and check whether responses are in line with the instructions. Checks were performed on all output to see whether the change mechanism descriptions in the output were identical to those included in the prompt. Doing so revealed instances of hallucination in which ChatGPT altered brief descriptions in the output generated after lengthier prompts. Where hallucinations were identified, ChatGPT was reprompted with the instructions followed by only those change mechanisms that were incorrectly processed. Furthermore, postprocessing involved minor adjustments to clean category and domain names in which the full label was not reported. In some conversations in which coding was directly into the TDF, the output showed instances in which >1 domain was allocated per change mechanism. In these cases, the first of the annotated domains was selected for use in data analysis. Finally, the clean data sets were aggregated by coding scheme version. This entailed creating 1 spreadsheet per coding scheme where the first column reflected the mechanism unique identifier, the second column reflected the brief descriptions, and the remaining 10 columns reflected the category or domain name allocated to the change mechanism by the conversation in which the respective coding scheme was applied.

### Quality Evaluation

Results from the preparation phase were presented as number of change mechanisms identified in the subset and number that ChatGPT correctly identified against human coding. We also present the total number of change mechanisms identified in the full data set across each of the conversations. Results from the development of the inductive and unconstrained deductive coding schemes were presented as number of categories or domains identified and median and range of label word count per coding scheme version. Furthermore, the content of the labels was inspected to check whether coding schemes were in line with the instructions (eg, no overlap in categories or labels). The structured deductive approach was not evaluated on any of these metrics as the coding matrix contained all 14 TDF domains and lacked labels with domain definitions.

The reliability of the coding schemes was evaluated by calculating the Fleiss κ [50] for intercoder agreement with >2 coders. Specifically, intercoder agreement calculation was performed for each version of the coding scheme developed using ChatGPT and the unstructured coding matrix by comparing allocated codes in the aggregated data sets. κ statistics were calculated per coding scheme (ie, overall κ score) and per category (or domain) within the coding scheme (ie, category-specific κ score) to compare reliability across and within coding schemes. κ scores of <0 were interpreted as poor agreement, scores from 0.00 to 0.20 were interpreted as slight agreement, scores from 0.21 to 0.40 were interpreted as fair agreement, scores from 0.41 to 0.60 were interpreted as moderate agreement, scores from 0.61 to 0.80 were interpreted as substantial agreement, and scores from 0.81 to 1.00 were interpreted as almost perfect agreement [51].

Furthermore, frequency data were generated for each of the coding schemes and the coding matrix. To do so, the mode across the 10 ChatGPT coders per change mechanism was retrieved for each of the aggregated data sets (ie, the data sets that combined the annotated data from 10 conversations). This meant that frequency data reflected a majority agreement among the ChatGPT coders rather than a consensus decision-making approach. Thus, frequency tables were created per coding scheme after a decision-making approach where the final classification of the mechanisms was based on the mode of the annotated data per applied coding scheme. Frequency data and κ scores were obtained using Stata software (version 18.0; StataCorp).

## Results

### Evaluation of Preparation Phase

As shown in Table 1, the number of change mechanisms identified from forum posts varied across data sets (ie, the ChatGPT conversations from the data preparation phase), ranging from 571 to 623 condensed meaning units. Precision rates between ChatGPT and human coding ranged between 0.66 and 0.88. In total, 2 data sets yielded a precision of 0.88, which meant that a decision needed to be made on which one to bring forward to the next phase. We selected the data set that identified the larger number of change mechanisms based on the data subset (n=127) as this would enable a greater number of change mechanisms to be incorporated in the development of the coding schemes.

**Table 1:** Precision in identifying change mechanisms during the preparation phase.

Data set generated during data preparation	Change mechanisms identified in the full data set, n	Change mechanisms identified in the 20% data subset, n	Mechanisms correctly identified from the subset, n	Precision
Data set 1	584	128	111	0.87
Data set 2	587	119	105	0.87
Data set 3^a^	585	127	112	0.88
Data set 4	602	118	104	0.88
Data set 5	571	131	110	0.84
Data set 6	616	128	109	0.85
Data set 7	619	132	114	0.86
Data set 8	584	124	107	0.86
Data set 9	623	135	115	0.85
Data set 10	591	121	101	0.66

^a^Data set selected for the development of coding schemes.

### Evaluation of the Inductive Approach

The results of the inductive approach are presented in Table 2. The inductively developed coding schemes contained a variable number of categories, ranging from 5 to 13. The category labels were overall lengthier than instructed, with a median label word count of 15 to 51 across coding schemes. Overall κ scores indicated substantial or almost perfect intercoder agreement for all coding schemes. Most definitions included specific examples of change mechanisms. Categories with labels reflecting a mixed or residual group of change mechanisms had category-specific κ scores indicating moderate or less than moderate intercoder agreement. Frequency tables based on a majority agreement decision-making approach showed that categories reflecting substituting sugary products (16%-29%) and gradually reducing sugar consumption (12%-20%) and a joint category reflecting both substituting and reducing sugar (41%) were the most frequently mentioned categories in the final annotated data sets. Multimedia Appendix 2 provides a detailed overview of all inductively developed coding schemes, related metrics, and frequency tables.

**Table 2:** Intercoder agreement for the application of the coding schemes from the inductive approach.

Aggregated data sets by version of data-driven coding scheme	Categories in coding scheme, n	Label word count, median (range)	Intercoder agreement, overall κ^a^
Version 1	10	21 (15-25)	0.76
Version 2	9	26 (13-34)	0.69
Version 3	5	28 (24-29)	0.79
Version 4	10	30 (21-36)	0.84
Version 5	9	15 (12-20)	0.77
Version 6	6	28 (23-36)	0.83
Version 7	10	25 (19-31)	0.77
Version 8	10	16 (11-22)	0.72
Version 9	13	51 (10-69)	0.77
Version 10	5	17 (15-19)	0.72

^a^*P*<.001 for intercoder agreement for all versions of the coding scheme.

Coding scheme 4 had the best intercoder agreement overall (κ=0.84; *P*<.001) and was selected for further evaluation. As indicated in Table 3, intercoder agreement on the categories within this coding scheme was almost perfect (ie, κ>0.80; *P*<.001) for all but 2 categories. Particularly high category-specific intercoder agreement (κ=0.91; *P*<.001) was observed for a category that included specification of what was not included in the category (ie, “This approach contrasts with gradual reduction or moderation strategies”). There was overlap in the names of 2 categories (ie, “Sugar alternatives and substitutes” and “Substitution and replacement approaches”); however, the category definitions clearly delineated these categories, and both categories had almost perfect intercoder agreement (κ=0.84; *P*<.001).

**Table 3:** Inductively developed coding scheme with best overall intercoder agreement.

Category	Definition	Category-specific intercoder agreement, κ^a^	Frequency distribution—change mechanisms coded into the category^b^ (n=585), n (%)
Psychological and behavioral strategies	Employing various psychological and behavioral techniques to change sugar consumption, such as seeking support, addressing addiction, recognizing the problem, planning ahead, and finding alternative distractions.	0.83	116 (19.8)
Substitution and replacement approaches	Replacing sugary foods and drinks with healthier alternatives, including options without added sugars; high-fiber carbs; fruits; nuts; or drinks like cinnamon tea, black coffee, or soda water.	0.84	111 (19)
Gradual reduction and moderation methods	Gradually reducing sugar consumption over time by reducing portion sizes, gradually decreasing sugar in tea/coffee, or incorporating a gradual process for adapting to reduced sugar. Moderation and small daily changes are emphasized.	0.85	85 (14.5)
Knowledge- and awareness-based approaches	Gaining knowledge about the harmful effects of excessive sugar consumption, checking sugar content in products, reading articles for information, and being aware of hidden sugars in various food products. Seeking resources and advice is also encouraged.	0.87	65 (11.1)
Environmental and practical strategies	Implementing changes in the environment to support reduced sugar consumption, such as changing grocery shopping habits, keeping cabinets stocked with healthy snacks, discarding carbs-filled foods, and not buying sugary items in the first place.	0.88	51 (8.7)
Health and well-being focus	Emphasizing the benefits of reduced sugar consumption on energy and overall health, incorporating nutrient-rich foods, ensuring adequate sleep, exercising to reduce stress, and considering health consequences and diabetes complications as motivation.	0.72	40 (6.8)
Elimination and cold turkey approaches	Completely quitting sugar consumption abruptly or going “cold turkey” for better health. This approach contrasts with gradual reduction or moderation strategies.	0.91	35 (6)
Support and community engagement	Seeking parental guidance, useful advice, moral support, weight loss buddies, or support from others. Sharing success stories and helping others break addiction are also emphasized.	0.95	30 (5.1)
Sugar alternatives and substitutions	Exploring and using various sugar alternatives and substitutes, including natural sweeteners such as stevia, honey, or Swerve, as well as incorporating naturally sweet options such as fruit.	0.84	28 (4.8)
Personal determination and accountability	Making a firm decision to quit sugar consumption, acknowledging weak moments and impulsive behavior, taking small steps, acknowledging the time and trial-and-error process, building willpower, and using tools such as MyFitnessPal to track sugar intake.	0.67	24 (4.1)

^a^*P*<.001 for intercoder agreement for all categories of the coding scheme.

^b^On the basis of a majority agreement decision-making approach across ChatGPT coders (ie, by selecting the mode of the codes per change mechanism).

We also examined the content of coding scheme version 2, which had the poorest overall intercoder agreement (κ=0.69; *P*<.001). The poorest category-specific agreement (κ=0.46; *P*<.001) within this coding scheme was for a miscellaneous category. Furthermore, overlap was observed in the definitions, with similar examples provided for multiple categories. For example, “seeking advice and support,” “seeking support from others,” and “seeking parental guidance” appeared in 3 different categories, and “avoiding purchasing sugary foods” and “changing shopping habits” appeared in 2 different categories.

### Evaluation of the Unconstrained Deductive Approach

Results from the unconstrained deductive approach are presented in Table 4. None of the developed TDF coding schemes contained all 14 domains. The number of domains identified from the data subset was variable, ranging from 6 to 10. Definition word counts were in line with the prompt instructions for all coding schemes (median between 12 and 17), with maximum definition word counts mostly being close to 20 words. The extent to which there was intercoder agreement overall was moderate or substantial across coding schemes.

**Table 4:** Intercoder agreement for the application of the coding schemes from the unconstrained deductive approach.

Aggregated data sets by version of theory-driven coding scheme	Domains, n	Label word count, median (range)	Overall intercoder agreement, κ^a^
Version 1	10	16 (12-19)	0.58
Version 2	8	13 (12-18)	0.62
Version 3	7	14 (13-17)	0.52
Version 4	7	16 (14-24)	0.73
Version 5	10	12 (9-13)	0.73
Version 6	10	15 (14-22)	0.53
Version 7	7	12 (11-19)	0.53
Version 8	9	14 (13-17)	0.62
Version 9	6	17 (13-19)	0.60
Version 10	10	16 (12-22)	0.58

^a^*P*<.001 for intercoder agreement for all versions of the coding scheme.

Across all TDF coding schemes, domain-specific κ scores ranged from 0.06 to 0.89 (Multimedia Appendix 3). The domains “Beliefs about consequences,” “Environmental context and resources,” and “Social influences” were identified in all versions of the coding scheme, whereas the domains “Optimism,” “Reinforcement,” and “Social and professional role and identity” were identified in none. The domain “Social influence” had the highest domain-specific intercoder agreement (κ=0.79-0.89; *P*<.001), whereas the domain “Memory, attention, and decision processes” had the lowest domain-specific intercoder agreement (κ=0.06-0.63; *P*<.001). There was no overlap in domain labels within coding schemes. Multimedia Appendix 4 provides a detailed overview of all TDF coding schemes, related metrics, and frequency tables. Of the coding schemes with the highest intercoder agreement (κ=0.73; *P*<.001), coding scheme 5 had the greatest number of TDF domains and, therefore, was selected for further evaluation. As indicated in Table 5, domain-specific intercoder agreement on 6 out of 10 domains within this coding scheme was substantial or near perfect.

**Table 5:** Unconstrained deductive coding scheme with best overall intercoder agreement.

Domain of the Theoretical Domains Framework	Definition	Domain-specific intercoder agreement, κ^a^	Frequency distribution—change mechanisms coded into the domain^b^ (n=585), n (%)
Behavioral regulation	Implementing strategies, techniques, and habits to regulate and control sugar consumption.	0.77	369 (63.1)
Knowledge	Acquiring information and understanding the effects of sugar on health and nutrition.	0.79	54 (9.2)
Beliefs about consequences	Understanding and acknowledging the positive outcomes of reducing sugar consumption motivate behavior change.	0.75	48 (8.2)
Social influences	External factors and support from others influence sugar consumption and dietary choices.	0.87	38 (6.5)
Environmental context and resources	The impact of the physical environment and available resources on sugar consumption habits.	0.61	34 (5.8)
Beliefs about capabilities	Confidence in one’s ability to change sugar consumption habits and overcome addiction.	0.57	24 (4.1)
Goals	Setting specific targets and objectives related to reducing sugar intake.	0.68	10 (1.7)
Emotion	Emotional factors that influence sugar cravings and behavior change.	0.35	5 (0.9)
Intention	Having a clear purpose and determination to change sugar consumption behavior.	0.59	3 (0.5)
Memory, attention, and decision processes	Cognitive processes that involve memory, attention, and decision-making in relation to sugar consumption.	0.13	0 (0)

^a^*P*<.001 for intercoder agreement for all domains of the coding scheme.

^b^On the basis of a majority agreement decision-making approach across ChatGPT coders (ie, by selecting the mode of the codes per change mechanism).

We compared domain labels across the coding schemes with the best and poorest intercoder agreement. This revealed that domains with better domain-specific intercoder agreement included examples of change mechanisms in their labels. An example of this was observed for the domain “Behavioral regulation,” labeled as “The self-directed process of monitoring, controlling, and modifying behaviors related to sugar consumption” in one version (κ=0.43; *P*<.001) and more descriptively labeled as “Techniques and strategies used to regulate and control sugar consumption, including portion control, substitution, gradual reduction, and self-monitoring” in another version (κ=0.77; *P*<.001).

### Evaluation of the Structured Deductive Approach

This approach used a structured coding matrix in which change mechanisms were coded directly into TDF domains without first specifying domain labels. This meant that, instead of 10 different coding schemes each applied using 10 different ChatGPT conversations, the structured deductive approach applied the single published coding matrix using 10 different conversations. The overall κ scores for the coding matrix indicated substantial intercoder agreement (κ=0.66; *P*<.001). As indicated in Table 6, domain-specific intercoder agreement on 7 domains of the TDF coding matrix had substantial or near-perfect agreement, and 4 domains had moderate agreement. The highest domain-specific intercoder agreement was observed for the domain “Social influences” (κ=0.85; *P*<.001), which similarly yielded almost perfect intercoder agreement in all but one of the unconstrained coding schemes (as per the previous section). The lowest domain-specific intercoder agreement was observed for the domain “Optimism” (κ=0.33; *P*<.001), which was not featured in any of the unconstrained coding schemes, and for the domain “Emotion” (κ=0.35; *P*=.55), which yielded fair to substantial intercoder agreement in the unconstrained deductively developed coding schemes.

**Table 6:** Intercoder agreement on the structured deductive approach using the Theoretical Domains Framework (TDF) coding matrix.

TDF domain	Domain-specific intercoder agreement, κ^a^	Frequency distribution—change mechanisms coded into the domain^b^ (n=585), n (%)
Behavioral regulation	0.66	281 (48)
Beliefs about consequences	0.73	82 (14)
Environmental context and resources	0.56	82 (14)
Knowledge	0.79	46 (7.9)
Social influences	0.85	29 (5)
Memory, attention, and decision processes	0.50	19 (3.2)
Social and professional role and identity	0.73	18 (3.1)
Beliefs about capabilities	0.54	12 (2.1)
Goals	0.74	7 (1.2)
Emotion	0.36^c^	4 (0.7)
Intentions	0.85	2 (0.3)
Optimism	0.33	2 (0.3)
Skills	0.46	1 (0.2)
Reinforcement	0	0 (0)

^a^*P*<.001 for intercoder agreement for all domains of the coding scheme unless otherwise stated.

^b^On the basis of a majority agreement decision-making approach across ChatGPT coders (ie, by selecting the mode of the codes per change mechanism).

^c^*P*=.51.

## Discussion

### Principal Findings

This study is among the first to use ChatGPT to automate a range of tasks related to qualitative content analysis of web-based data on behavior change. Preparation for the analysis process was done by identifying relevant change mechanisms from forum data on reducing sugar consumption, which we did through ChatGPT with an estimated 88% precision rate. On the basis of a subset of change mechanisms, 10 coding schemes were developed using an inductive approach in which categories and category labels were informed by the data. Another 10 coding schemes were developed using an unconstrained deductive approach categories that reflected relevant domains of the TDF and were relabeled in line with the current data on sugar reduction. The developed coding schemes largely followed prompt instructions but were highly variable in the number of categories across and within approaches. Using ChatGPT to code the full change mechanism data set into each coding scheme showed moderate to almost perfect intercoder agreement, where the intercoder agreement of the inductively developed coding schemes was generally superior to that of the deductively developed coding schemes. A structured deductive approach was also applied by coding directly into the original TDF coding matrix without specifying domain labels to reflect the current data set. The overall intercoder agreement for this approach exceeded that for most coding schemes from the unconstrained deductive approach, but it was lower than the overall agreement observed for the inductively developed coding schemes.

### Comparison With Other Studies

There have been a few exploratory studies using ChatGPT to analyze small data sets with analysis approaches that bear similarities to the inductive and deductive content analysis approaches used in our study. For example, one study used ChatGPT to conduct reflexive thematic analysis on a feature newspaper article about post–COVID-19 condition [35]. Analysis of the text was data driven as ChatGPT was prompted to generate a list of categories featured in the data, which, in contrast to the approach in our study, was then compared to a list of categories independently developed by a researcher. The findings revealed that the lists were largely similar, although the ChatGPT-generated list included more focused categories than the broader human-developed categories. Both lists were combined to refine the categories, and ChatGPT was queried to confirm the fit of the refined categories with the data, reflective of a negotiation step when combining category lists. Other research has used ChatGPT to assess problem-solving through content analysis of 40 short internet chats in which university students aimed to solve a mathematical issue [33]. Using an inductive approach, the authors prompted ChatGPT to generate a list of categories with description (comparable to the coding schemes in our study) for each chat and provide an overall problem-solving score based on the extent to which the categories were featured in the chat. The reliability of ChatGPT output over time was evaluated by repeating exact prompts at a different date in new conversations, which revealed only a moderately positive correlation between the output at different time points. Our study showed more promising results as we observed substantial to almost perfect intercoder agreement when evaluating the reliability of the inductively developed coding schemes by repeating prompts in conversations across time points and OpenAI accounts.

The aforementioned data from problem-solving chats were also analyzed using a deductive approach [33]. To do so, the authors prompted ChatGPT with questions reflecting categories of a theory-driven coding scheme that had previously been applied by researchers to code the data. Overall problem-solving scores were calculated from the number of categories that were featured in a chat. Prompts were repeated at 2 time points to calculate intracoder agreement. The findings showed 90% intracoder agreement in output generated on separate occasions. Our study similarly evaluated the reliability of ChatGPT output by repeating prompts in conversations across time points and OpenAI accounts, albeit across 10 conversations as opposed to 2. Interestingly, our findings were more promising for the inductive approach, whereas we observed only moderate to substantial overall agreement for the coding schemes from the unconstrained deductive approach.

The deductive approach with a structured coding matrix in our study is somewhat similar to the approach taken in another study that evaluated feedback on a university course [34]. The study used ChatGPT to code 200 student comments directly into a predefined list of categories that were based on the literature but not accompanied by a label to define the categories. Researchers subsequently double checked the ChatGPT-annotated data and agreed with 85% of the allocated codes. An alternative double check was performed by prompting ChatGPT to rate how well an allocated category reflected the content of the comment. Approximately 10% of the annotated data received low ratings, suggesting incorrectly coded data. These findings do not necessarily indicate an error in ChatGPT coding; rather, they highlight specific categories of the coding matrix that might need to be refined.

Our objective was to explore the utility of ChatGPT in conducting qualitative content analysis with an emphasis on diminishing the human workload and time spent on analysis tasks and the error prone–ness related to human fatigue when analyzing large data sets. In line with this, human input in our study was focused on prompt engineering and data preparation, whereas the inductive and deductive analysis was conducted by prompting ChatGPT with a set of instructions related to those tasks. As such, it was beyond our scope to combine ChatGPT-generated coding schemes with a human-developed coding scheme or compare ChatGPT-annotated data with human-annotated data. However, as we used secondary data, it is possible to compare findings related to our secondary aim with the change mechanisms identified in the original study that relied solely on manual coding [41]. The original study identified 25 different categories that were organized into 4 phases of readiness to change. The most frequent categories were similar to the inductive approach categories “Substance substitution,” “Knowledge and information,” and “Avoidance.” However, the most frequently occurring category in the inductive and deductive approaches was related to behavioral regulation, which the previous study coded into smaller categories that reflected the study’s overarching theory and coding scheme.

### Implications and Future Directions

Our study suggests that intercoder agreement using the inductive approach was superior to that achieved through deductive approaches. This finding aligns with the fundamental principles underlying each approach as the inductive approach, developed solely on the current data set, aims to describe its content. This approach is particularly valuable in novel or emerging areas of study or when preexisting data, concepts, or theories are scarce [3]. In fields such as behavior change, numerous explanatory and predictive models and theories offer insights into not only what is happening but also why. Our study indicates that coding within a model or theory yields higher intercoder agreement when the coding scheme is tailored to reflect the current data set. However, adjusting a published coding matrix poses the risk of missing opportunities to advance widely applicable models such as the TDF, which has demonstrated universal utility [39,52]. Moreover, as a framework, the TDF is more explanatory than predictive, and some of its categories overlap (eg, planning appears in 2 different categories), complicating data analysis methods such as content analysis [53]. Future research may benefit from applying the insights from our study to behavior change theories characterized by more clearly defined categories, such as the theory of planned behavior, to further explore ChatGPT utility.

The difference between intercoder agreement for inductive and deductive approaches might be further explained by prompt engineering and category development used by ChatGPT. For example, category labels from the inductive approach mostly exceeded the maximum word count specified in the instructions but also commonly included examples, which was not the case for the labels from the deductive unconstrained approach. LLMs process data based on semantic relations in text, and as illustrated by the literature [54], queries with richer semantic data (eg, synonyms) increase LLM performance. Examples included in the category labels are likely semantically related to the short descriptions of change mechanisms, which might, in turn, result in more consistent annotation of the data. Future research is warranted to confirm whether coding schemes that include examples increase intercoder reliability across ChatGPT output.

Our findings underline the importance of including a step in the analysis process in which coding schemes are refined based on data that are to be coded as opposed to using a structured coding matrix. Depending on the topic investigated, certain elements of a theory or framework may be less applicable to the data but indicate new insights into the topic [3]. This may explain why domains that were not featured in the unconstrained coding schemes showed low domain-specific agreement when applying the structured coding matrix. Without the option to code into domains not featured in the data subset, the overall intercoder agreement using this approach may have been better. A noteworthy issue with the structured deductive approach was that, despite instructions to only code mechanisms into the best-matching domain, there were still considerable data across coding schemes allocated to more than one domain. The issue persisted despite adapting instructions for the task during the prompt engineering phase. This could be an artifact of the underlying TDF knowledge, which in some settings results in an overlap between domains [53]. It might also be that, with the lack of domain labels and, thus, limited semantic information, instructions are more ambiguous, thereby leaving more room for interpretation and resulting in less consistent output across conversations.

Through a thorough process of prompt engineering, we developed a set of structured prompts that can easily be adapted, expanded, and tailored by other researchers intending to use ChatGPT for qualitative content analysis. The extent to which prompts need adaptation and further prompt engineering depends on the research topic and approach. Replicating this study on a different topic would require an adequate description of that topic, which might be limited to a substitution of the topic keywords used in the prompts (ie, “sugar consumption” and “change mechanisms”). Where other theories are selected for the inductive approach, it is advisable to check ChatGPT’s familiarity with the theory. Depending on the topic and theory, we suggest specifying instructions with other notes on what to look out for during task execution. The use of appropriate synonyms is also recommended to improve ChatGPT performance [54] as terminology may vary across research groups, disciplines, and communities. With other versions of ChatGPT, token limit may not be a restriction, and we suggest that researchers experiment with adding more context to the prompt. To further automate the preparation task, comparison with human coding may be replaced by having ChatGPT rate the accuracy of previous output, as done in previous research [34].

The approaches used in this study can be tested on other data types, such as survey responses, interviews, or focus group discussions. However, the nature of the data (eg, depth vs breadth and level of structuredness) might affect ChatGPT performance on analysis tasks, and research is warranted to refine prompt engineering and assess the reliability of the output based on other data types. Regardless of the research approach, when using tools such as ChatGPT for qualitative analysis, researchers should be aware of ethical considerations to prevent harm, such as transparency, informed consent, data privacy, and potential biases in LLM training data or disseminated results [55,56]. It is warranted that such issues are considered properly to ensure that research projects abide by ethical standards.

It should be reiterated that our study focused primarily on the reliability of ChatGPT-annotated data without addressing the validity of the findings. Assessing the validity of the findings is an important area for future investigation. Validity checks could be incorporated through triangulation of investigators, for instance, by having experienced qualitative researchers develop coding schemes and compare these with coding schemes developed using ChatGPT. Alternatively, the prompt with step-by-step instructions, which we used to create a theory-driven coding scheme, enables a less time-intensive method to check the validity of the ChatGPT-generated coding scheme. In this type of prompt, the first part of the output facilitates the possibility to assess whether the data underlying each category in the generated coding scheme accurately reflect the categories. In addition, we encourage others using ChatGPT for qualitative content analysis to experiment with coding rules that may further increase the quality of the output and prevent overlap between categories. It should also be mentioned that we presented frequency data based on the coding schemes with the best intercoder agreement. However, the selection of the most optimal coding scheme may depend on other considerations, including expert reflection of categories and labels generated by ChatGPT, number of categories within a coding scheme, and the need to incorporate certain categories or mix inductive and deductive approaches. Moreover, the appropriateness of the analysis methods and techniques used is dependent on the type of topic as well as the character and volume of the available data [19].

### Limitations

The colloquial phrase “garbage in, garbage out” can be seen as a general rule in machine learning, meaning that any output retrieved is only as good as the data underlying the output [57]. As we followed a thorough process of prompt engineering and evaluation of the output did not reveal concerning deviations from the prompt instructions, we are relatively confident that the quality of the output was not substantially diminished by the instructions for the tasks. However, it should be noted that sourcing the data to be analyzed from web-based forums may have implications for the quality of the output. Forum data are user generated and, by nature, less focused than data generated though targeted questions (eg, as is the case with interview and survey data), potentially leading to convoluted data [58]. In our study, this issue was partly circumvented by using secondary data that met certain eligibility criteria to ensure that they related to lived experiences of people trying to change sugar consumption [41]. Still, using these data to generate a condensed set of change mechanisms did result in the inclusion of nontarget data, as evident from our estimate of 88% precision in identification of change mechanisms, which we considered acceptable for use in the inductive and deductive analysis approaches.

Whether precision levels should be considered acceptable is dependent on the goal of the study and is often a trade-off with other metrics of model performance, including recall and accuracy [59]. When generating a data set of change mechanisms identified from the forum posts, we focused on precision to optimize the amount of relevant data to be used in creating and applying the coding schemes. Imperfect precision could have led to distorted categories in a coding scheme as instructions specified to group all mechanisms, regardless of relevance, in discreet categories. It may also have caused the creation of a miscellaneous category, observed in various versions of the inductively developed coding schemes. Similarly, coding nontarget change mechanisms into the coding schemes may have distorted the frequency data as these were also based on all data. We included instructions to code change mechanisms as not applicable in cases in which they did not fit into any category, and these data could be further examined to build new knowledge of the TDF and how it applies to a range of different contexts.

### Conclusions

AI assistance has the potential to make a massive impact on research involving qualitative content analysis. As demonstrated in this study, ChatGPT can assist with each phase of the methodology, from condensing data and developing coding schemes to double coding and facilitating consensus meetings. While we have shown that ChatGPT can perform these tasks largely without human oversight, there are many risks associated with this approach, with the key risk being that the output may reflect the worst of ChatGPT’s coding and, therefore, may not accurately reflect the data or research questions. We recommend that human involvement is necessary, but it could be reduced to just 1 or 2 researchers across each phase. Such human involvement would largely ensure that prompt engineering and interpretation remain aligned with the research goals and context. This study provides the foundations for qualitative content analysis using ChatGPT and can be tested and further developed as new AI emerges.

## Appendix

Multimedia Appendix 1 Final prompts.

Multimedia Appendix 2 Inductively developed coding schemes and metadata.

Multimedia Appendix 3 Domain-specific κ scores per the Theoretical Domains Framework coding scheme.

Multimedia Appendix 4 Deductively developed coding schemes and metadata.

## Abbreviations

AI: artificial intelligence
LLM: large language model
TDF: Theoretical Domains Framework

## Data Availability

The data sets generated and analyzed during this study are available from the corresponding author on reasonable request.
