# Supplementary material for: ChatGPT for Automated Qualitative Research: Content Analysis
Source: J Med Internet Res. 2024 Jul 25;26:e59050. doi: 10.2196/59050 (PMC11310599; doi:10.2196/59050)
Supplement: Multimedia Appendix 1 [file jmir_v26i1e59050_app1.docx]

## **Multimedia Appendix 1: Final prompts**

### Data Preparation

Prompt 1: Creating dataset of condensed meaning units

| Below are forum posts reflecting real-life experiences related to changing sugar consumption. For each of these posts, please extract all potential mechanisms/strategies/techniques for reducing sugar consumption. Each mechanism should be described in a brief and concise manner, using up to 10 words. If multiple mechanisms can be extracted from a single post, each should be mentioned separately. Please note that mechanisms may not be explicitly presented as such, so be sure to interpret and extract them from the forum posts accordingly.  First format the response as:  “  Post 1:  - mechanism 1: {max 10 word description}  - mechanism 2: {max 10 word description}  - etc (if applicable)  Post 2:  - mechanism 1: {max 10 word description}  - mechanism 2: {max 10 word description}  - etc (if applicable)  ”  Then please reformat the response as an excel spreadsheet with in the first column the post number, the second the mechanism number, and the third the mechanism description. Please format the table as csv and put the table in code block.  Forum posts:  ""  *[insert forum posts]*  "" |
| --- |

### Inductive Approach: Development and Application of Data-Driven Coding Schemes

Prompt 2: Developing data-driven coding scheme

| Below are descriptions of mechanisms related to changing sugar consumption. Please carefully analyse and compare the provided mechanisms to identify patterns, themes, or principles that align and differentiate the mechanisms. Then cluster the mechanisms into meaningful groups reflecting their shared underlying pattern/theme/principle to change sugar consumption. Each group should be discrete and distinct, with no overlap between the groups. Please provide a brief definition of the shared underlying pattern of each cluster, using up to 20 words.  Format your response as:  “  Group 1: {underlying pattern cluster 1}.  Definition: {max 20-word definition cluster 1}  Group 2: {underlying pattern cluster 2}.  Definition: {max 20-word definition cluster 2}  ”  Mechanism descriptions:  ""  *[insert list of mechanism descriptions based on 20% data subset]*  "" |
| --- |

Prompt 3: Applying data-driven coding scheme

| Below are descriptions of mechanisms related to changing sugar consumption. Based on the provided descriptions, can you make an excel spreadsheet with in the first column the mechanism number and the second the mechanism description? Can you also add a third column in which you classify each mechanism under one of the predefined mechanism groups listed below using the mechanism group definition. Please use the best matching mechanism group. Each mechanism should be listed in only one mechanism group. If a mechanism can be classified under more than one mechanism group, please choose the best matching one. If the mechanism description does not fit under any of the predefined mechanism groups add ‘N/A’ to the third column. Please format the table as csv and put the table in code block.    Predefined mechanism groups:  *[insert list of mechanism groups from response to previous prompt – post processing required: extracting only names of mechanism groups from response]*  Mechanism group definitions:  *[insert full response to previous prompt – post processing may be required to insert text in response format as specified in previous prompt]*  Strategy descriptions:  ""  *[insert numbered list of mechanisms from condensation task,* ***full dataset****]*  "" |
| --- |

#### **Unconstrained Deductive Approach: Development and Application of Theory-Driven Coding Schemes**

Prompt 4: Developing unconstrained coding scheme informed by the TDF

| Below are descriptions of mechanisms related to changing sugar consumption. Please carefully analyse and compare the provided mechanisms to identify patterns, themes, or principles that align with the domains of the “Theoretical Domains Framework” (i.e., knowledge; skills; social/professional role and identity; beliefs about capabilities; optimism; beliefs about consequences; reinforcement; intentions; goals; memory, attention and decision process; environmental context and resources; social influences; emotion; behavioural regulation). Cluster the mechanisms into groups that reflect TDF domains. Each group should reflect only one domain. Your answer should list all mechanisms under the best aligning domain. Each mechanism should be listed only once.  First format the response as:  “  {TDF domain name}: ({mechanism #}, {mechanism #} etc.)  {TDF domain name}: ({mechanism #}, {mechanism #} etc.)  ”  Then provide a definition for each domain based on the underlying mechanism descriptions, using up to 20 words. Where a domain was not identified in the first part of your response, give “N/A” as definition.  Format second part of your response as:  “  {TDF domain name}.  Definition: {max 20-word definition related to underlying mechanisms}  {TDF domain name}.  Definition: {max 20-word definition related to underlying mechanisms}  etc.  ”  Mechanism descriptions:  ""  *[insert list of mechanism descriptions based on 20% data subset]*  "" |
| --- |

Prompt 5: Applying unconstrained coding scheme informed by the TDF

| Below are descriptions of mechanisms related to changing sugar consumption. Based on the provided descriptions, can you make an excel spreadsheet with in the first column the mechanism number and the second the mechanism description? Can you also add a third column in which you classify each mechanism under one of the predefined mechanism groups listed below using the mechanism group definition. Please use the best matching mechanism group. Each mechanism should be listed in only one mechanism group. If a mechanism can be classified under more than one mechanism group, please choose the best matching one. If the mechanism description does not fit under any of the predefined mechanism groups add ‘N/A’ to the third column. Please format the table as csv and put the table in code block.  Predefined mechanism groups:  *[insert list of mechanism groups from response to previous prompt – post processing required: extracting only names of mechanism groups from response]*  Mechanism group definitions:  *[insert full response to previous prompt – post processing may be required to insert text in response format as specified in previous prompt]*  Strategy descriptions:  ""  *[insert numbered list of mechanisms from condensation task,* ***full dataset****]*  "" |
| --- |

#### **Structured Deductive Approach: Coding Directly into TDF Coding Matrix**

Prompt 6: Classifying directly into TDF domains using coding matrix

| Below are descriptions of mechanisms related to changing sugar consumption. Based on the provided descriptions, can you make an excel spreadsheet with in the first column the mechanism number and the second the mechanism description? Can you also add a third column in which you classify each strategy under one of the Theoretical Domains Framework (TDF) domains listed below. Please use the best matching TDF domain. If a mechanism can be classified under more than one TDF domain, please choose the best matching one. Please format the table as csv and put the table in code block.  TDF domains:  knowledge; skills; social/professional role and identity; beliefs about capabilities; optimism; beliefs about consequences; reinforcement; intentions; goals; memory, attention and decision process; environmental context and resources; social influences; emotion; behavioural regulation  Mechanism descriptions:  ""  [insert numbered list of mechanisms from condensation task, **full dataset**]  "" |
| --- |
