# Supplementary material for: ChatGPT for Automated Qualitative Research: Content Analysis
Source: J Med Internet Res. 2024 Jul 25;26:e59050. doi: 10.2196/59050 (PMC11310599; doi:10.2196/59050)
Supplement: Multimedia Appendix 2 [file jmir_v26i1e59050_app2.docx]

## **Multimedia Appendix 2: Inductively developed coding schemes and meta data**

**Table S1** Inductive coding scheme version 1 (overall kappa=0.76; *P*<.001)

| **Category** | **Category label**  **(i.e., definition)** | **Label word count** | **Inter-coder kappa^a^** | **N(%)^b^** |
| --- | --- | --- | --- | --- |
| Awareness and Education. | Understanding the harmful effects of excessive sugar consumption, checking labels, seeking information, and acknowledging the problem to drive behavioral change. | 20 | 0.79 | 64 (10.9) |
| Behavioral Strategies for Sugar Reduction. | Implementing various behavioral techniques, such as seeking support, planning ahead, and making gradual changes, to reduce sugar consumption. | 18 | 0.58 | 75 (12.8) |
| Cold Turkey Approach. | Completely eliminating sugar consumption abruptly, avoiding sugary foods and drinks altogether for better health outcomes. | 15 | 0.83 | 22 (3.8) |
| External Factors and Environment. | Manipulating the external environment to reduce sugar consumption, such as changing grocery shopping habits, keeping healthy snacks stocked, and removing sugary foods from the house. | 25 | 0.82 | 51 (8.7) |
| Gradual Reduction and Moderation. | Gradually reducing sugar intake over time, moderating portion sizes, practicing moderation, and making small daily changes to achieve long-term success. | 20 | 0.78 | 84 (14.4) |
| Health Consciousness. | Considering health consequences, aiming for better energy and overall health, prioritizing nutrient-rich foods, and making conscious choices to reduce sugar intake. | 21 | 0.54 | 19 (3.2) |
| Mindset and Motivation. | Adopting a positive mindset, giving oneself credit, recognizing weak moments, and building willpower and determination to overcome sugar cravings and addiction. | 21 | 0.73 | 59 (10.1) |
| Specific Dietary Approaches. | Following specific diets like low carb (Atkins or Keto), alkaline-balancing foods, or The Maker's Diet to control sugar intake and improve overall health. | 23 | 0.69 | 36 (6.2) |
| Substitution and Alternative Choices. | Replacing sugary foods and drinks with healthier alternatives, such as fruits, nuts, high-fiber carbs, or sugar-free options. | 17 | 0.85 | 140 (23.9) |
| Support and Accountability. | Seeking guidance, advice, and support from others, including parental guidance, weight loss buddies, and online resources, to stay motivated and accountable in reducing sugar consumption. | 25 | 0.90 | 34 (5.8) |

^a^*P*<.001 for intercoder agreement for all categories of the coding scheme; ^b^Frequency table of allocated codes on full dataset of change mechanisms.

**Table S2** Inductive coding scheme version 2 (overall kappa=0.69; *P*<.001)

| **Category** | **Category label**  **(i.e., definition)** | **Label word count** | **Inter-coder kappa^a^** | **N(%)^b^** |
| --- | --- | --- | --- | --- |
| Cognitive and Behavioral Strategies to Change Sugar Consumption. | Implementing various cognitive and behavioral techniques to modify sugar consumption habits and cravings, such as seeking parental guidance, addressing addiction, finding strategies to stop cravings, and changing shopping habits. | 29 | 0.58 | 145 (24.8) |
| Education and Awareness of Sugar's Effects on Health. | Understanding the harmful effects of excessive sugar consumption, acknowledging the problem, seeking advice and support, checking sugar content in products, and learning about hidden sugars in various foods. | 28 | 0.85 | 61 (10.4) |
| Environmental Modification and Supportive Measures for Sugar Reduction. | Making changes to the physical environment, such as keeping a cabinet stocked with healthy snacks, discarding sugary foods, avoiding purchasing them, and seeking support from others. | 26 | 0.79 | 49 (8.4) |
| Gradual Reduction and Moderation Approaches for Sugar Consumption. | Gradually reducing sugar portions, decreasing sugar in tea/coffee over time, practicing moderation, and making small daily changes to achieve long-term success. | 21 | 0.73 | 94 (16.1) |
| Individualized Techniques and Mindset Shifts for Sugar Reduction. | Developing personal strategies to resist cravings, recognizing weak moments, planning ahead, acknowledging time and trial, setting goals, giving oneself credit, and focusing on the joy of friendship rather than artificial joy from sugar. | 33 | 0.12 | 3 (0.5) |
| Medical and Supplemental Support for Sugar Reduction. | Seeking medical advice, incorporating supplements like boron, and using natural sweeteners like stevia. | 13 | 0.75 | 22 (3.8) |
| Other Miscellaneous Techniques for Sugar Reduction. | Various additional techniques that do not fit into the other clusters, such as using dark blue dishes, avoiding red and yellow colors while eating, practicing structured eating, and using MyFitnessPal to track sugar intake. | 34 | 0.46 | 34 (5.8) |
| Specific Dietary Approaches to Reduce Sugar Consumption. | Following specific diets or meal plans, such as low carb diets (Atkins or Keto), alkaline-balancing foods, The Maker's Diet, and focusing on nutrient-rich, unprocessed foods. | 25 | 0.69 | 40 (6.8) |
| Substitution and Replacement Techniques for Sugar Consumption. | Substituting sugary foods and drinks with healthier alternatives, incorporating foods without added sugars, and finding alternative drinks or snacks to reduce sugar intake. | 23 | 0.82 | 137 (23.4) |

^a^*P*<.001 for intercoder agreement for all categories of the coding scheme; ^b^Frequency table of allocated codes on full dataset of change mechanisms.

**Table S3** Inductive coding scheme version 3 (overall kappa=0.79; *P*<.001)

| **Category** | **Category label**  **(i.e., definition)** | **Label word count** | **Inter-coder kappa^a^** | **N(%)^b^** |
| --- | --- | --- | --- | --- |
| Dietary and Nutritional Approaches to Reduce Sugar Consumption. | Focusing on dietary changes and nutritional strategies to reduce sugar intake, such as consuming protein-rich foods, incorporating high-fiber carbohydrates, choosing low-sugar alternatives, and emphasizing whole, unprocessed foods. | 27 | 0.64 | 74 (12.6) |
| Health Education and Awareness for Sugar Consumption. | Increasing knowledge and awareness about the negative effects of excessive sugar consumption, learning to identify hidden sugars in food products, and making informed choices based on health consequences. | 28 | 0.82 | 67 (11.5) |
| Psychological and Behavioral Strategies to Change Sugar Consumption. | Employing various psychological and behavioral techniques to modify sugar consumption habits, such as seeking support, recognizing and managing cravings, setting goals, and replacing sugary foods with healthier alternatives. | 28 | 0.83 | 172 (29.4) |
| Substitution and Gradual Reduction Techniques for Sugar Consumption. | Employing methods that involve substituting sugary foods and drinks with healthier alternatives, gradually reducing sugar intake over time, and making conscious choices to avoid purchasing or consuming high-sugar products. | 29 | 0.82 | 240 (41) |
| Support and Accountability for Sugar Consumption Reduction. | Seeking support from others, either through friends, family, or weight loss buddies, to stay motivated and accountable in the journey to reduce sugar consumption. | 24 | 0.88 | 32 (5.5) |

^a^*P*<.001 for intercoder agreement for all categories of the coding scheme; ^b^Frequency table of allocated codes on full dataset of change mechanisms.

**Table S4** Inductive coding scheme version 4 (overall kappa=0.84; *P*<.001)

| **Category** | **Category label**  **(i.e., definition)** | **Label word count** | **Inter-coder kappa^a^** | **N(%)^b^** |
| --- | --- | --- | --- | --- |
| Elimination and Cold Turkey Approaches. | Completely quitting sugar consumption abruptly or going "cold turkey" for better health. This approach contrasts with gradual reduction or moderation strategies. | 21 | 0.91 | 35 (6) |
| Environmental and Practical Strategies. | Implementing changes in the environment to support reduced sugar consumption, such as changing grocery shopping habits, keeping cabinets stocked with healthy snacks, discarding carb-filled foods, and not buying sugary items in the first place. | 34 | 0.88 | 51 (8.7) |
| Gradual Reduction and Moderation Methods. | Gradually reducing sugar consumption over time, either by reducing portion sizes, gradually decreasing sugar in tea/coffee, or incorporating a gradual process for adapting to reduced sugar. Moderation and small daily changes are emphasized. | 33 | 0.85 | 85 (14.5) |
| Health and Well-being Focus. | Emphasizing the benefits of reduced sugar consumption on energy and overall health, incorporating nutrient-rich foods, ensuring adequate sleep, exercising to reduce stress, and considering health consequences and diabetes complications as motivation. | 31 | 0.72 | 40 (6.8) |
| Knowledge and Awareness-Based Approaches. | Gaining knowledge about the harmful effects of excessive sugar consumption, checking sugar content in products, reading articles for information, and being aware of hidden sugars in various food products. Seeking resources and advice is also encouraged. | 36 | 0.87 | 65 (11.1) |
| Personal Determination and Accountability. | Making a firm decision to quit sugar consumption, acknowledging weak moments and impulsive behavior, taking small steps, acknowledging the time and trial-and-error process, building willpower, and using tools like MyFitnessPal to track sugar intake. | 34 | 0.67 | 24 (4.1) |
| Psychological and Behavioral Strategies. | Employing various psychological and behavioral techniques to change sugar consumption, such as seeking support, addressing addiction, recognizing the problem, planning ahead, and finding alternative distractions. | 25 | 0.83 | 116 (19.8) |
| Substitution and Replacement Approaches. | Replacing sugary foods and drinks with healthier alternatives, including options without added sugars, high fiber carbs, fruits, nuts, or drinks like cinnamon tea, black coffee, or soda water. | 28 | 0.84 | 111 (19) |
| Sugar Alternatives and Substitutes. | Exploring and utilizing various sugar alternatives and substitutes, including natural sweeteners like stevia, honey, or Swerve, as well as incorporating naturally sweet options like fruit. | 25 | 0.84 | 28 (4.8) |
| Support and Community Engagement. | Seeking parental guidance, useful advice, moral support, weight loss buddies, or support from others. Sharing success stories and helping others break addiction are also emphasized. | 25 | 0.95 | 30 (5.1) |

^a^*P*<.001 for intercoder agreement for all categories of the coding scheme; ^b^Frequency table of allocated codes on full dataset of change mechanisms.

**Table S5** Inductive coding scheme version 5 (overall kappa=0.77; *P*<.001)

| **Category** | **Category label**  **(i.e., definition)** | **Label word count** | **Inter-coder kappa^a^** | **N(%)^b^** |
| --- | --- | --- | --- | --- |
| Avoidance and Elimination. | Eliminating or avoiding sugary foods, drinks, and environments to prevent temptation and reduce sugar consumption. | 15 | 0.81 | 69 (11.8) |
| Behavioral Modification Strategies. | Employing various tactics and approaches to modify behavior and habits associated with sugar consumption. | 14 | 0.57 | 55 (9.4) |
| Education and Awareness. | Gaining knowledge about the detrimental effects of excessive sugar consumption and raising awareness to make informed choices. | 17 | 0.86 | 69 (11.8) |
| External Support and Accountability. | Seeking guidance, support, and motivation from others to successfully change sugar consumption habits. | 13 | 0.89 | 38 (6.5) |
| Gradual Reduction Approach. | Implementing a step-by-step reduction strategy to gradually decrease sugar intake over time. | 12 | 0.85 | 72 (12.3) |
| Health-Oriented Approaches. | Focusing on overall health improvement, including adopting nutrient-rich diets, incorporating exercise, and ensuring adequate sleep. | 15 | 0.69 | 54 (9.2) |
| Mindfulness and Self-Control. | Cultivating mindfulness, recognizing triggers, and exercising self-control to resist cravings and make conscious choices regarding sugar consumption. | 17 | 0.53 | 37 (6.3) |
| Personal Motivation and Determination. | Developing a strong personal drive and commitment to change, relying on willpower, and setting specific goals to reduce sugar consumption. | 20 | 0.69 | 43 (7.4) |
| Substitution and Alternatives. | Replacing sugary foods and drinks with healthier alternatives or substitutes to reduce sugar intake. | 14 | 0.87 | 148 (25.3) |

^a^*P*<.001 for intercoder agreement for all categories of the coding scheme; ^b^Frequency table of allocated codes on full dataset of change mechanisms.

**Table S6** Inductive coding scheme version 6 (overall kappa=0.83; *P*<.001)

| **Category** | **Category label**  **(i.e., definition)** | **Label word count** | **Inter-coder kappa^a^** | **N(%)^b^** |
| --- | --- | --- | --- | --- |
| Education and Awareness for Sugar Consumption Change. | Increasing knowledge about the harmful effects of excessive sugar consumption, understanding hidden sugars in various food products, checking sugar content in items, and seeking resources to make informed choices. | 29 | 0.90 | 54 (9.2) |
| Environmental and Behavioral Adjustments for Sugar Consumption Change. | Making changes to the physical and social environment to support reduced sugar consumption, such as modifying grocery shopping habits, keeping healthier snacks stocked, avoiding trigger foods, and eliminating or limiting access to sugary items at home. | 36 | 0.80 | 69 (11.8) |
| Gradual Reduction and Incremental Changes for Sugar Consumption Change. | Implementing a step-by-step approach to gradually reduce sugar intake, whether through small daily changes, portion control, decreasing sugar in beverages over time, or adopting a systematic process. | 27 | 0.82 | 83 (14.2) |
| Health-Focused Approaches for Sugar Consumption Change. | Emphasizing the health benefits of reducing sugar consumption, incorporating exercise to distract from cravings, prioritizing sleep for better control over sugar and processed food cravings, and considering diabetes complications as motivation to reduce sugar intake. | 35 | 0.73 | 60 (10.3) |
| Psychological Strategies for Sugar Consumption Change. | Utilizing various psychological techniques, such as seeking support, recognizing the problem, practicing moderation, and adopting a positive approach, to change sugar consumption habits. | 23 | 0.87 | 155 (26.5) |
| Substitution and Alternative Approaches to Sugar Consumption Change. | Replacing sugary foods and drinks with healthier alternatives, incorporating nutrient-rich options, finding substitutes for sugar cravings, and exploring alternative recipes to reduce sugar consumption. | 24 | 0.86 | 164 (28) |

^a^*P*<.001 for intercoder agreement for all categories of the coding scheme; ^b^Frequency table of allocated codes on full dataset of change mechanisms.

**Table S7** Inductive coding scheme version 7 (overall kappa=0.77; *P*<.001)

| **Category** | **Category label**  **(i.e., definition)** | **Label word count** | **Inter-coder kappa^a^** | **N(%)^b^** |
| --- | --- | --- | --- | --- |
| Awareness and education about the effects of sugar consumption. | Understanding the harmful effects of excessive sugar intake, hidden sugars in food products, and the role of nutrient-rich foods in reducing cravings for sugary items. | 25 | 0.86 | 61 (10.4) |
| Behavioral strategies for managing sugar consumption. | Implementing various tactics, such as seeking support, planning, recognizing triggers, and making gradual changes, to modify sugar consumption habits. | 19 | 0.62 | 108 (18.5) |
| Elimination and avoidance strategies for sugar reduction. | Completely cutting out sugar (cold turkey), avoiding sugary foods and drinks, not purchasing them, and removing them from the home to reduce access and temptation. | 25 | 0.90 | 64 (10.9) |
| Gradual reduction and moderation approaches to sugar intake. | Gradually reducing sugar portions, decreasing sugar in beverages gradually, implementing moderation, allowing occasional indulgences, and practicing portion control to manage sugar consumption. | 22 | 0.79 | 72 (12.3) |
| Mindset and motivation for changing sugar consumption. | Shifting mindset, acknowledging the problem, giving oneself credit, adopting a positive approach, and finding motivation in health benefits, addiction management, or diabetes complications. | 23 | 0.70 | 59 (10.1) |
| Specific dietary plans or methods for reducing sugar. | Following specific diets or approaches like the Maker's Diet, low-carb diets (Atkins or Keto), structured eating (happy stomach tactic), or focusing on alkaline-balancing or whole, unprocessed foods. | 27 | 0.71 | 20 (3.4) |
| Specific tactics and techniques to combat sugar cravings. | Employing specific methods to address cravings, such as distraction techniques (taking baths, chewing gum), consuming certain foods (cinnamon tea, almonds, fruit), or modifying habits related to sleep, exercise, or grocery shopping. | 31 | 0.69 | 25 (4.3) |
| Substitutions and alternatives for reducing sugar intake. | Replacing sugary foods and beverages with healthier options, such as low-sugar or sugar-free alternatives, fruits, high-fiber carbs, and natural sweeteners. | 20 | 0.84 | 133 (22.7) |
| Support systems and external resources for sugar reduction. | Utilizing external sources of support, including parental guidance, medical advice, online articles, weight loss buddies, and resources like apps and websites to aid in sugar reduction. | 26 | 0.87 | 35 (6) |
| Unique or miscellaneous approaches to reducing sugar consumption. | Utilizing unconventional methods such as color association (avoiding red and yellow colors while eating, eating on dark blue dishes), boron supplementation, or using Facebook videos for identifying sugar in foods. | 30 | 0.59 | 8 (1.4) |

^a^*P*<.001 for intercoder agreement for all categories of the coding scheme; ^b^Frequency table of allocated codes on full dataset of change mechanisms.

**Table S8** Inductive coding scheme version 8 (overall kappa=0.72; *P*<.001)

| **Category** | **Category label**  **(i.e., definition)** | **Label word count** | **Inter-coder kappa^a^** | **N(%)**^b^ |
| --- | --- | --- | --- | --- |
| Addiction management and overcoming cravings for sugar. | Addressing sugar and junk food addiction, managing cravings, and finding alternative ways to cope with cravings for sugary items. | 19 | 0.75 | 36 (6.2) |
| Avoidance and elimination of sugary foods and drinks. | Taking proactive steps to avoid purchasing, consuming, or keeping sugary items at home to eliminate temptations and reduce sugar intake. | 20 | 0.74 | 60 (10.3) |
| Behavioral strategies for sugar consumption change. | Implementing various behavioral techniques and strategies to modify sugar consumption habits. | 11 | 0.56 | 77 (13.2) |
| Education and awareness of sugar consumption and its consequences. | Understanding the harmful effects of excessive sugar consumption and making informed choices based on knowledge. | 15 | 0.84 | 67 (11.5) |
| Gradual reduction and moderation in sugar consumption. | Adopting a gradual approach to reducing sugar intake, practicing moderation, and making small, sustainable changes over time. | 17 | 0.72 | 95 (16.2) |
| Health-focused lifestyle changes for reducing sugar consumption. | Incorporating various lifestyle changes, such as exercise, adequate sleep, and nutritious food choices, to support the reduction of sugar consumption. | 20 | 0.69 | 60 (10.3) |
| Personal commitment and mindset shift for sugar reduction. | Making a firm decision to quit or reduce sugar consumption, acknowledging the need for change, and adopting a positive mindset for success. | 22 | 0.65 | 30 (5.1) |
| Substituting sugary foods and beverages with healthier alternatives. | Replacing high-sugar foods and drinks with lower-sugar or sugar-free options to reduce overall sugar intake. | 15 | 0.76 | 96 (16.4) |
| Substitution of artificial sweeteners and natural sweeteners. | Using alternative sweeteners, such as stevia or honey, to replace sugar in food and beverages. | 15 | 0.84 | 31 (5.3) |
| Support and accountability in changing sugar consumption. | Seeking guidance, support, and accountability from others to successfully modify sugar consumption habits. | 13 | 0.84 | 32 (5.5) |

^a^*P*<.001 for intercoder agreement for all categories of the coding scheme; ^b^Frequency table of allocated codes on full dataset of change mechanisms.

**Table S9** Inductive coding scheme version 9 (overall kappa=0.77; *P*<.001)

| **Category** | **Category label**  **(i.e., definition)** | **Label word count** | **Inter-coder kappa^a^** | **N(%)^b^** |
| --- | --- | --- | --- | --- |
| Behavior Modification and Habit Formation. | Address sugar and junk food addiction, find strategies to stop sugar and junk food cravings, acknowledge and recognize the problem, adopt a positive approach to dealing with addictions, recognize weak moments and impulsive sugar consumption, identify trigger foods and avoid them, practice moderation in sugar consumption, incorporate cheat days for special occasions, build willpower to resist sugary drinks, habituation and not having soda at home. | 65 | 0.57 | 39 (6.7) |
| Behavioral Conditioning Techniques. | Avoid red and yellow colors while eating, eat on dark navy blue or blue dishes. | 15 | 0.55 | 2 (0.3) |
| Education and Awareness of Sugar Consumption. | Understand the harmful effects of excessive sugar consumption, check sugar content in products and choose lower options, explore sugar-free and gluten-free recipes, read free articles for more information, be aware of hidden sugars in various food products, watch a video on identifying sugar in foods, consider health consequences of sugar consumption. | 51 | 0.89 | 61 (10.4) |
| Environmental Modification and Avoidance. | Change grocery shopping habits to avoid sugary foods, discard carb-filled foods and avoid buying more, cut out excess sugar from sweets and other sources, limit purchases of soft drinks to reduce access, don't buy soft drinks for home, get rid of sugary foods from the house, keep junk food out of the house, avoid buying sugary items at the grocery store. | 61 | 0.89 | 44 (7.5) |
| Gradual Reduction and Moderation Approaches. | Gradually reduce sugar portions when craving, gradually reduce sugar in tea/coffee over time, gradually decrease sugar in tea/coffee by small amounts, gradually reduce sugar in tea/coffee without notice, gradually reduce sugar in coffee, gradually eliminate white sugar from the diet, transition to stevia gradually, master moderation instead of eliminating sugar entirely, four-step process to cut down sugar. | 57 | 0.81 | 115 (19.7) |
| Healthy Lifestyle Choices and Substitutions. | Consume cinnamon tea to reduce sugar cravings, keep a cabinet stocked with healthy snack options, consume protein in the morning and every meal, replace sugar's artificial joy with the joy of friendship, eat nutrient-rich foods reduces taste for super-sweet foods, exercise to reduce stress and distract from cravings, eat alkaline-balancing foods to reduce cravings, supplement with boron to reduce sugar dependence. | 61 | 0.75 | 70 (12) |
| Mindfulness and Self-awareness. | Acknowledge it takes time and trial and error, measure daily intake with apps like "Myfitnesspal," write "No Sugar January" to remind oneself daily, take small steps and let them 'take root,' determine portion size and stick to it, be cautious of concentrated fruit juices in foods, ensure meals are adequately filling to avoid snacking, eat only when hungry. | 58 | 0.62 | 14 (2.4) |
| Professional Assistance and Medical Advice. | Seek medical advice, diabetes complications as motivation to reduce sugar. | 10 | 0.88 | 15 (2.6) |
| Psychological and Emotional Approaches. | Give yourself credit for deciding to fix the issue, replace sugar's artificial joy with the joy of friendship, take it slow, but believe in your ability to succeed, help others break addiction with success. | 34 | 0.69 | 55 (9.4) |
| Sleep and Stress Management. | Sufficient sleep reduces sugar and processed food cravings, use sleep as a strategy to resist sugar cravings, exercise to reduce stress and distract from cravings. | 25 | 0.83 | 5 (0.9) |
| Specific Dietary Approaches. | Low carb diet (Atkins or Keto), choose bread with high fiber and low sugar, eat whole, unprocessed foods and cook at home, follow The Maker's Diet for better health. | 29 | 0.81 | 25 (4.3) |
| Substitution and Replacement Strategies. | Replace sugary foods with options without added sugars, incorporate foods you enjoy but without added sugars, substitute sugary foods with high fiber carbs, satisfy sugar cravings with almonds or other nuts, replace sugary drinks with black coffee, replace Coke with soda water, choose chocolates with high cacao content and no sugar, substitute fruit for dessert or limit cake/pie portions, use stevia as a sweetener for cooked foods and beverages. | 69 | 0.85 | 103 (17.6) |
| Support and Guidance for Behavior Change. | Seek assistance from parents, seek useful advice, and moral support from others, plan ahead, and receive support from others, find a weight loss buddy for motivation and support. | 28 | 0.74 | 33 (5.6) |

^a^*P*<.001 for intercoder agreement for all categories of the coding scheme; ^b^Frequency table of allocated codes on full dataset of change mechanisms.

**Table S10** Inductive coding scheme version 10 (overall kappa=0.72; *P*<.001)

| **Category** | **Category label**  **(i.e., definition)** | **Label word count** | **Inter-coder kappa^a^** | **N(%)^b^** |
| --- | --- | --- | --- | --- |
| Behavioral Strategies for Sugar Reduction. | Utilizing various behavioral techniques and practical measures to modify habits and cravings associated with sugar consumption. | 16 | 0.67 | 182 (31.1) |
| Education and Knowledge for Sugar Reduction. | Gaining information and understanding about the effects of excessive sugar consumption, reading resources, and seeking advice for better decision-making. | 19 | 0.85 | 65 (11.1) |
| Mindset and Awareness for Sugar Reduction. | Focusing on mindset shifts, awareness of sugar-related issues, and adopting a positive approach towards reducing sugar consumption. | 17 | 0.76 | 93 (15.9) |
| Specific Techniques and Approaches for Sugar Reduction. | Employing specific methods and strategies, such as sleep regulation, gradual reduction, and utilizing external tools, to reduce sugar intake. | 19 | 0.50 | 75 (12.8) |
| Substitutes and Alternatives for Sugar Reduction. | Introducing healthier substitutes and alternative options to satisfy cravings and reduce reliance on sugary foods. | 15 | 0.81 | 170 (29.1) |

^a^*P*<.001 for intercoder agreement for all categories of the coding scheme; ^b^Frequency table of allocated codes on full dataset of change mechanisms.
