# Supplementary material for: ChatGPT for Automated Qualitative Research: Content Analysis
Source: J Med Internet Res. 2024 Jul 25;26:e59050. doi: 10.2196/59050 (PMC11310599; doi:10.2196/59050)
Supplement: Multimedia Appendix 3 [file jmir_v26i1e59050_app3.docx]

## **Multimedia Appendix 3: Domain-specific kappa scores per TDF coding scheme**

**Table S1.** Domain-specific kappa scores per TDF coding scheme^a^

| TDF domain | Version of TDF coding scheme | | | | | | | | | |
| --- | --- | --- | --- | --- | --- | --- | --- | --- | --- | --- |
|  | **V1** | **V2** | **V3** | **V4** | **V5** | **V6** | **V7** | **V8** | **V9** | **V10** |
|  |  |  |  |  |  |  |  |  |  |  |
| Behavioural regulation | 0.63 | 0.64 | 0.43 | 0.77 | 0.77 | 0.58 | x | x | 0.61 | 0.62 |
| Beliefs about capabilities | 0.35 | x | x | x | 0.57 | 0.41 | 0.47 | 0.63 | x | 0.47 |
| Beliefs about consequences | 0.62 | 0.67 | 0.62 | 0.66 | 0.75 | 0.60 | 0.72 | 0.73 | 0.65 | 0.60 |
| Emotion | 0.65 | 0.51 | 0.54 | 0.64 | 0.35 | 0.57 | x | 0.57 | 0.62 | 0.47 |
| Environmental context and resources | 0.59 | 0.56 | 0.67 | 0.74 | 0.61 | 0.54 | 0.60 | 0.62 | 0.50 | 0.44 |
| Goals | 0.52 | x | x | 0.58 | 0.68 | 0.54 | 0.47 | 0.45 | x | 0.59 |
| Intentions | 0.46 | x | x | x | 0.59 | x | x | 0.41 | x | 0.44 |
| Knowledge | x | 0.73 | x | 0.78 | 0.79 | 0.72 | x | x | x | 0.67 |
| Memory, attention, and decision processes | 0.63 | 0.17 | 0.36 | x | 0.13 | 0.06 | 0.48 | 0.35 | 0.48 | 0.07 |
| Optimism | x | x | x | x | x | x | x | x | x | x |
| Reinforcement | x | x | x | x | x | x | x | x | x | x |
| Skills | 0.23 | 0.30 | 0.42 | x | x | 0.41 | 0.33 | 0.63 | x | x |
| Social influences | 0.85 | 0.83 | 0.79 | 0.83 | 0.87 | 0.80 | 0.89 | 0.85 | 0.84 | 0.81 |
| Social/professional role and identity | x | x | x | x | x | x | x | x | x | x |

^a^*P*<.001 for intercoder agreement for all domains of the TDF coding scheme; x = kappa could not be calculated as this domain was not identified in coding scheme.
