# Supplementary material for: ChatGPT for Automated Qualitative Research: Content Analysis
Source: J Med Internet Res. 2024 Jul 25;26:e59050. doi: 10.2196/59050 (PMC11310599; doi:10.2196/59050)
Supplement: Multimedia Appendix 4 [file jmir_v26i1e59050_app4.docx]

## **Multimedia Appendix 4: Deductively developed coding schemes and meta data**

**Table S1.** Unconstrained TDF coding scheme version 1 (overall kappa=0.58; *P*<.001)

| **Category**  **(i.e., TDF domain)** | **Category label**  **(i.e., definition)** | **Label word count** | **Inter-coder kappa^a^** | **N(%)^b^** |
| --- | --- | --- | --- | --- |
| Behavioural regulation | Implementing strategies for gradually reducing sugar portions, substituting sugary foods, or finding alternative drinks. | 14 | 0.63 | 325 (55.6) |
| Beliefs about consequences | Understanding and recognizing the harmful effects of excessive sugar consumption on energy and overall health. | 15 | 0.62 | 55 (9.4) |
| Environmental context and resources | Utilizing external resources, such as websites, articles, and apps, for information, tracking, and support in changing sugar consumption habits. | 19 | 0.59 | 48 (8.2) |
| Emotion | Acknowledging emotions, celebrating progress, and maintaining a positive approach while dealing with sugar addiction. | 14 | 0.65 | 44 (7.5) |
| Social influences | Seeking guidance, support, and advice from others to change sugar consumption habits. | 12 | 0.85 | 38 (6.5) |
| Memory, attention, and decision processes | Being mindful of trigger foods, checking sugar content, reading labels, and making conscious decisions about sugar intake. | 17 | 0.63 | 29 (5) |
| Goals | Setting objectives and milestones, breaking down the process into small steps, and giving oneself credit for progress. | 17 | 0.52 | 21 (3.6) |
| Beliefs about capabilities | Adopting a positive mindset, believing in one's ability to overcome addiction, and recognizing weak moments and impulsive behaviors. | 18 | 0.35 | 11 (1.9) |
| Intentions | Acknowledging the problem and making a firm decision to reduce or quit sugar consumption. | 14 | 0.46 | 8 (1.4) |
| Skills | Developing strategies, such as changing grocery shopping habits or stocking healthy snack options, to avoid sugary foods. | 17 | 0.23 | 6 (1) |

^a^*P*<.001 for intercoder agreement for all domains of the coding scheme; ^b^Frequency table of allocated codes on full dataset of change mechanisms.

**Table S2.** Unconstrained TDF coding scheme version 2 (overall kappa=0.62; *P*<.001)

| **Category**  **(i.e., TDF domain)** | **Category label**  **(i.e., definition)** | **Label word count** | **Inter-coder kappa^a^** | **N(%)^b^** |
| --- | --- | --- | --- | --- |
| Behavioural regulation | The ability to self-regulate behavior, set goals, and implement strategies to achieve desired outcomes. | 14 | 0.64 | 340 (58.1) |
| Beliefs about consequences | Perceptions of the positive and negative outcomes or effects of a behavior. | 12 | 0.67 | 66 (11.3) |
| Environmental context and resources | Physical and social surroundings, as well as available tools and support, that can facilitate or hinder behavior change. | 18 | 0.56 | 59 (10.1) |
| Knowledge | Awareness or understanding of facts, information, and concepts relevant to a behavior. | 12 | 0.73 | 52 (8.9) |
| Social influences | The influence of other people on an individual's thoughts, feelings, and behavior. | 12 | 0.83 | 29 (5) |
| Emotion | The affective state or feeling experienced by an individual that can influence behavior. | 13 | 0.51 | 22 (3.8) |
| Skills | Proficiency or ability to perform specific actions or tasks related to a behavior. | 13 | 0.30 | 16 (2.7) |
| Memory, attention, and decision processes | Cognitive processes involved in storing, retrieving, and using information to make decisions and take action | 15 | 0.17 | 1 (0.2) |

^a^*P*<.001 for intercoder agreement for all domains of the coding scheme; ^b^Frequency table of allocated codes on full dataset of change mechanisms.

**Table S3.** Unconstrained TDF coding scheme version 3 (overall kappa=0.52; *P*<.001)

| **Category**  **(i.e., TDF domain)** | **Category label**  **(i.e., definition)** | **Label word count** | **Inter-coder kappa^a^** | **N(%)^b^** |
| --- | --- | --- | --- | --- |
| Skills | Acquiring and applying specific techniques, strategies, or actions to change sugar consumption habits. | 13 | 0.42 | 209 (35.7) |
| Behavioural regulation | The self-directed process of monitoring, controlling, and modifying behaviors related to sugar consumption. | 13 | 0.43 | 149 (25.5) |
| Beliefs about consequences | Perceptions of the positive or negative outcomes and impacts associated with changing sugar consumption. | 14 | 0.62 | 77 (13.2) |
| Environmental context and resources | The physical and social circumstances, supports, and aids that can influence sugar consumption and behavior change. | 16 | 0.67 | 71 (12.1) |
| Emotion | The role of feelings and affective states in the process of changing sugar consumption. | 14 | 0.54 | 33 (5.6) |
| Social influences | The impact of social relationships, norms, and support on sugar consumption behavior change. | 13 | 0.79 | 32 (5.5) |
| Memory, attention, and decision processes | Cognitive functions and mental activities involved in remembering, paying attention, and making choices related to sugar consumption. | 17 | 0.36 | 14 (2.4) |
| Skills | Acquiring and applying specific techniques, strategies, or actions to change sugar consumption habits. | 13 | 0.42 | 209 (35.7) |

^a^*P*<.001 for intercoder agreement for all domains of the coding scheme; ^b^Frequency table of allocated codes on full dataset of change mechanisms.

**Table S4.** Unconstrained TDF coding scheme version 4 (overall kappa=0.73; *P*<.001)

| **Category**  **(i.e., TDF domain)** | **Category label**  **(i.e., definition)** | **Label word count** | **Inter-coder kappa^a^** | **N(%)^b^** |
| --- | --- | --- | --- | --- |
| Behavioural regulation | Techniques and strategies used to regulate and control sugar consumption, including portion control, substitution, gradual reduction, and self-monitoring. | 18 | 0.77 | 337 (57.6) |
| Beliefs about consequences | Awareness and understanding of the positive and negative outcomes associated with changing sugar consumption. | 14 | 0.66 | 62 (10.6) |
| Environmental context and resources | The physical and social environment in which sugar consumption occurs, including access to healthy foods, grocery shopping habits, and availability of support and resources. | 24 | 0.74 | 53 (9.1) |
| Knowledge | Acquiring and understanding information about sugar consumption, its effects, and strategies to reduce intake. | 14 | 0.78 | 50 (8.5) |
| Emotion | The emotional aspects involved in managing sugar consumption, such as dealing with cravings, stress, and distraction techniques. | 17 | 0.64 | 33 (5.6) |
| Social influences | The impact of social interactions, support systems, and role models on sugar consumption and behavior change. | 16 | 0.83 | 32 (5.5) |
| Goals | Setting specific objectives and intentions to modify sugar consumption habits and achieve desired outcomes. | 14 | 0.58 | 18 (3.1) |

^a^*P*<.001 for intercoder agreement for all domains of the coding scheme; ^b^Frequency table of allocated codes on full dataset of change mechanisms.

**Table S5.** Unconstrained TDF coding scheme version 5 (overall kappa=0.73; *P*<.001)

| **Category**  **(i.e., TDF domain)** | **Category label**  **(i.e., definition)** | **Label word count** | **Inter-coder kappa^a^** | **N(%)^b^** |
| --- | --- | --- | --- | --- |
| Behavioural regulation | Implementing strategies, techniques, and habits to regulate and control sugar consumption. | 11 | 0.77 | 369 (63.1) |
| Knowledge | Acquiring information and understanding about the effects of sugar on health and nutrition. | 13 | 0.79 | 54 (9.2) |
| Beliefs about consequences | Understanding and acknowledging the positive outcomes of reducing sugar consumption motivate behavior change. | 13 | 0.75 | 48 (8.2) |
| Social influences | External factors and support from others influence sugar consumption and dietary choices. | 12 | 0.87 | 38 (6.5) |
| Environmental context and resources | The impact of the physical environment and available resources on sugar consumption habits. | 13 | 0.61 | 34 (5.8) |
| Beliefs about capabilities | Confidence in one's ability to change sugar consumption habits and overcome addiction. | 12 | 0.57 | 24 (4.1) |
| Goals | Setting specific targets and objectives related to reducing sugar intake. | 10 | 0.68 | 10 (1.7) |
| Emotion | Emotional factors that influence sugar cravings and behavior change. | 9 | 0.35 | 5 (0.9) |
| Intentions | Having a clear purpose and determination to change sugar consumption behavior. | 11 | 0.59 | 3 (0.5) |
| Memory, attention, and decision processes | Cognitive processes that involve memory, attention, and decision-making in relation to sugar consumption. | 13 | 0.13 | (0) |

^a^*P*<.001 for intercoder agreement for all domains of the coding scheme; ^b^Frequency table of allocated codes on full dataset of change mechanisms.

**Table S6.** Unconstrained TDF coding scheme version 6 (overall kappa=0.53; *P*<.001)

| **Category**  **(i.e., TDF domain)** | **Category label**  **(i.e., definition)** | **Label word count** | **Inter-coder kappa^a^** | **N(%)^b^** |
| --- | --- | --- | --- | --- |
| Behavioural regulation | Mechanisms focused on strategies and actions employed to regulate and modify sugar consumption behaviors. | 14 | 0.58 | 351 (60) |
| Knowledge | Mechanisms associated with acquiring, understanding, and utilizing information and knowledge about sugar consumption and its impact on health. | 18 | 0.72 | 52 (8.9) |
| Environmental context and resources | Mechanisms related to the physical environment, resources, and external factors that influence sugar consumption and can be modified to facilitate behavior change. | 22 | 0.54 | 50 (8.5) |
| Beliefs about consequences | Mechanisms reflecting the understanding and beliefs about the effects and outcomes of reducing sugar consumption. | 15 | 0.60 | 46 (7.9) |
| Social influences | Mechanisms influenced by the support, advice, and guidance of others in relation to changing sugar consumption. | 16 | 0.80 | 35 (6) |
| Emotion | Mechanisms related to the positive or negative emotional experiences associated with changing sugar consumption. | 14 | 0.57 | 26 (4.4) |
| Beliefs about capabilities | Mechanisms reflecting individuals' beliefs in their own abilities to successfully change their sugar consumption habits. | 15 | 0.41 | 12 (2.1) |
| Goals | Mechanisms related to setting specific objectives or targets in the process of reducing sugar consumption. | 15 | 0.54 | 12 (2.1) |
| Skills | Mechanisms related to the development and application of specific abilities and competencies to facilitate changes in sugar consumption habits. | 19 | 0.41 | 1 (0.2) |
| Memory, attention, and decision processes | Mechanisms involving cognitive processes such as memory, attention, and decision-making that influence sugar consumption behaviors. | 15 | 0.06 | (0) |

^a^*P*<.001 for intercoder agreement for all domains of the coding scheme; ^b^Frequency table of allocated codes on full dataset of change mechanisms.

**Table S7.** Unconstrained TDF coding scheme version 7 (overall kappa=0.53; *P*<.001)

| **Category**  **(i.e., TDF domain)** | **Category label**  **(i.e., definition)** | **Label word count** | **Inter-coder kappa^a^** | **N(%)^b^** |
| --- | --- | --- | --- | --- |
| Environmental context and resources | The impact of the physical and social environment, as well as the availability of resources, on sugar consumption behavior. | 19 | 0.6 | 255 (43.6) |
| Skills | Acquiring and developing abilities and strategies to effectively change sugar consumption behavior. | 12 | 0.33 | 123 (21) |
| Memory, attention, and decision processes | Cognitive processes involved in remembering information, paying attention to cues, and making decisions regarding sugar consumption behavior. | 17 | 0.48 | 64 (10.9) |
| Beliefs about consequences | Understanding the positive outcomes and benefits associated with reducing sugar consumption. | 11 | 0.72 | 63 (10.8) |
| Goals | Setting specific objectives and targets related to changing sugar consumption behavior. | 11 | 0.47 | 40 (6.8) |
| Social influences | The role of interpersonal relationships, social support, and social norms in influencing sugar consumption behavior. | 15 | 0.89 | 31 (5.3) |
| Beliefs about capabilities | Confidence in one's ability to change sugar consumption behavior and overcome challenges. | 12 | 0.47 | 9 (1.5) |

^a^*P*<.001 for intercoder agreement for all domains of the coding scheme; ^b^Frequency table of allocated codes on full dataset of change mechanisms.

**Table S8.** Unconstrained TDF coding scheme version 8 (overall kappa=0.62; *P*<.001)

| **Category**  **(i.e., TDF domain)** | **Category label**  **(i.e., definition)** | **Label word count** | **Inter-coder kappa^a^** | **N(%)^b^** |
| --- | --- | --- | --- | --- |
| Skills | Applying specific strategies and techniques related to changing sugar consumption and managing cravings. | 13 | 0.63 | 350 (59.8) |
| Environmental context and resources | The influence of the physical and social environment, as well as available resources, on sugar consumption behaviors. | 17 | 0.62 | 67 (11.5) |
| Beliefs about consequences | Understanding the positive impact of reducing sugar consumption on energy levels and overall health. | 14 | 0.73 | 60 (10.3) |
| Social influences | The impact of social interactions, support, and advice from others on changing sugar consumption habits. | 15 | 0.85 | 30 (5.1) |
| Emotion | Recognizing and managing the emotional aspects associated with sugar cravings and making behavioral changes. | 14 | 0.57 | 27 (4.6) |
| Intentions | Having the motivation and determination to modify sugar consumption habits and follow specific strategies. | 14 | 0.41 | 19 (3.2) |
| Goals | Setting targets and objectives related to reducing sugar intake and adopting healthier alternatives. | 13 | 0.45 | 14 (2.4) |
| Beliefs about capabilities | Having confidence in one's ability to change sugar consumption habits and control cravings. | 13 | 0.63 | 9 (1.5) |
| Memory, attention, and decision processes | The cognitive processes involved in remembering information, paying attention to cues, and making decisions regarding sugar consumption. | 17 | 0.35 | 9 (1.5) |

^a^*P*<.001 for intercoder agreement for all domains of the coding scheme; ^b^Frequency table of allocated codes on full dataset of change mechanisms.

**Table S9.** Unconstrained TDF coding scheme version 9 (overall kappa=0.60; *P*<.001)

| **Category**  **(i.e., TDF domain)** | **Category label**  **(i.e., definition)** | **Label word count** | **Inter-coder kappa^a^** | **N(%)^b^** |
| --- | --- | --- | --- | --- |
| Behavioural regulation | Implementing strategies and techniques to regulate and control behavior regarding sugar consumption, including planning, goal-setting, and self-monitoring. | 17 | 0.61 | 342 (58.5) |
| Environmental context and resources | Utilizing the physical and social environment, as well as available resources, to support and facilitate changes in sugar consumption. | 19 | 0.5 | 74 (12.6) |
| Beliefs about consequences | Recognizing and understanding the potential positive and negative outcomes of changing sugar consumption. | 13 | 0.65 | 71 (12.1) |
| Emotion | Considering and addressing the emotional aspects related to changing sugar consumption, such as stress, cravings, and enjoyment. | 17 | 0.62 | 38 (6.5) |
| Memory, attention, and decision processes | Engaging cognitive processes, including memory, attention, and decision-making, to consciously modify sugar consumption habits. | 14 | 0.48 | 31 (5.3) |
| Social influences | Seeking and leveraging social support and guidance from others in the process of changing sugar consumption. | 16 | 0.84 | 29 (5) |
| Behavioural regulation | Implementing strategies and techniques to regulate and control behavior regarding sugar consumption, including planning, goal-setting, and self-monitoring. | 17 | 0.61 | 342 (58.5) |
| Environmental context and resources | Utilizing the physical and social environment, as well as available resources, to support and facilitate changes in sugar consumption. | 19 | 0.5 | 74 (12.6) |
| Beliefs about consequences | Recognizing and understanding the potential positive and negative outcomes of changing sugar consumption. | 13 | 0.65 | 71 (12.1) |
| Emotion | Considering and addressing the emotional aspects related to changing sugar consumption, such as stress, cravings, and enjoyment. | 17 | 0.62 | 38 (6.5) |
| Memory, attention, and decision processes | Engaging cognitive processes, including memory, attention, and decision-making, to consciously modify sugar consumption habits. | 14 | 0.48 | 31 (5.3) |
| Social influences | Seeking and leveraging social support and guidance from others in the process of changing sugar consumption. | 16 | 0.84 | 29 (5) |

^a^*P*<.001 for intercoder agreement for all domains of the coding scheme; ^b^Frequency table of allocated codes on full dataset of change mechanisms.

**Table S10.** Unconstrained TDF coding scheme version 10 (overall kappa=0.58; *P*<.001)

| **Category**  **(i.e., TDF domain)** | **Category label**  **(i.e., definition)** | **Label word count** | **Inter-coder kappa^a^** | **N(%)^b^** |
| --- | --- | --- | --- | --- |
| Behavioural regulation | Using various strategies and techniques to modify behavior and habits related to sugar consumption. | 14 | 0.62 | 357 (61) |
| Beliefs about consequences | Considering the positive or negative outcomes of reducing sugar consumption on energy, health, and well-being. | 15 | 0.6 | 66 (11.3) |
| Knowledge | Having knowledge and understanding about the harmful effects of excessive sugar consumption and learning about alternatives and healthier options. | 19 | 0.67 | 52 (8.9) |
| Environmental context and resources | Considering the external factors and resources that can influence sugar consumption, such as grocery shopping habits and availability of healthy snack options. | 22 | 0.44 | 46 (7.9) |
| Social influences | Seeking support, guidance, and motivation from others to change sugar consumption habits. | 12 | 0.81 | 34 (5.8) |
| Emotion | Recognizing and managing emotions related to cravings, weak moments, and the process of reducing sugar consumption. | 16 | 0.47 | 16 (2.7) |
| Goals | Setting specific goals and intentions to reduce sugar consumption and work towards healthier habits. | 14 | 0.59 | 8 (1.4) |
| Beliefs about capabilities | Having confidence in one's ability to change sugar consumption habits and believing in personal competence and control. | 17 | 0.47 | 5 (0.9) |
| Intentions | Forming clear intentions and plans to reduce sugar consumption and committing to making changes. | 14 | 0.44 | 1 (0.2) |
| Memory, attention, and decision processes | Considering the cognitive processes involved in remembering, paying attention, and making decisions regarding sugar consumption and cravings. | 17 | 0.07 | (0) |

^a^*P*<.001 for intercoder agreement for all domains of the coding scheme; ^b^Frequency table of allocated codes on full dataset of change mechanisms.
